# Costs and cost-effectiveness of community health worker programs on reproductive, maternal, newborn and child health in low- and middle-income countries (2015–2024): A scoping review

**DOI:** 10.1371/journal.pgph.0004893

**Published:** 2026-01-22

**Authors:** Madelyn Miyares, Linnea Stansert Katzen, Kelsey Vaughan, Cleo Baskin, Madeleine Ballard, Maryse Kok, Ariwame Jimenez, M. Matias Iberico, Josef Ernst, Jessica Cook, Angele Bienvenue Ishimwe, Lily Martin, Patrick Kawooya, Zeus Aranda, Molly Mantus, Meghan Bruce Kumar, Karen E. Finnegan, Sandra Mudhune, Mardieh Dennis, Daniel Palazuelos, Dickson Nansima Mbewe, Michee Nshimayesu, Shobhana Nagraj, Rachel Vreeman, James O’Donovan

**Affiliations:** 1 Department of Health Policy and Management, Gillings School of Global Public Health, University of North Carolina at Chapel Hill, Chapel Hill, North Carolina, United States of America; 2 Community Health Impact Coalition, London, United Kingdom; 3 Institute for Life Course Health Research, Department of Global Health, Faculty of Medicine and Health Sciences, Stellenbosch University, Cape Town, South Africa; 4 Centre for Health and Sustainability, Department of Women’s and Children’s Health, Uppsala University, Uppsala, Sweden; 5 Bang for Buck Consulting, Amsterdam, Netherlands; 6 ResiliSUS Lab, Fundação Oswaldo Cruz, Rio de Janeiro, Brazil; 7 Arnhold Institute for Global Health, Icahn School of Medicine at Mount Sinai, New York, New York, United States of America; 8 Department of Clinical Sciences, Liverpool School of Tropical Medicine, Liverpool, United Kingdom; 9 Malawi-Liverpool Wellcome Research Programme, Blantyre, Malawi; 10 Compañeros En Salud, Ángel Albino Corzo, México; 11 Tulane University School of Medicine, New Orleans, Louisiana, United States of America; 12 Partners in Health, Boston, Massachusetts, United States of America; 13 TIP Global Health, Kigali, Rwanda; 14 NYU Health Sciences Library, NYU Langone Health, New York, New York, United States of America; 15 Nama Community Wellness Center, Mukono, Uganda; 16 Last Mile Health, Boston, Massachusetts, United States of America; 17 Department of Nursing, Midwifery and Health, Northumbria University, Newcastle upon Tyne, United Kingdom; 18 Department of Global Health and Social Medicine, Harvard Medical School, Boston, Massachusetts, United States of America; 19 Pivot, Ranomafana, Madagascar; 20 Lwala Community Alliance, Rongo, Kenya; 21 Last Mile Health, Monrovia, Liberia; 22 Division of Global Health Equity, Brigham and Women’s Hospital, Harvard Medical School, Boston, Massachusetts, United States of America; 23 Kasungu District Hospital, Kasungu, Malawi; 24 University of Global Health Equity, Kigali, Rwanda; 25 Department of Public Health and Primary Care, University of Cambridge, Cambridge, United Kingdom; 26 East London NHS Foundation Trust, London, United Kingdom; 27 Department of Global Health and Department of Pediatrics, Icahn School of Medicine at Mount Sinai, New York, New York, United States of America; University College London, UNITED KINGDOM OF GREAT BRITAIN AND NORTHERN IRELAND

## Abstract

Community Health Workers (CHWs) are vital in delivering primary health care in low- and middle-income countries (LMICs). To inform their broader rollout, this study updates a 2015 review, critically examining the costs, cost-effectiveness and affordability of reproductive, maternal, newborn and child health (RMNCH) CHW programs in LMICs. A scoping review was conducted using ten databases and grey literature, covering studies published between August 2015 and July 2024. Search terms related to “Community Health Workers” and “Economic Evaluations” were used. Studies were screened via Covidence software based on inclusion and exclusion criteria. Data on study methodology, costs, and outcomes were extracted, tabulated in Microsoft Excel, and analysed. Across 53 studies (21 about reproductive health, maternal and newborn care and 32 child health focused), covering 161 scenarios, the most common cost metrics for CHW-led interventions were cost per beneficiary (ranging from $0.02 to $1,547), cost per capita (ranging from $0.09 to $20.25), and cost per consultation (ranging from $0.26 to $52.91). Of 100 scenarios that assessed cost-effectiveness, the majority concluded CHWs were cost-effective, most frequently when compared against an alternative service or delivery modality, such as facility-based care, or the no-longer widely accepted threshold of a country’s gross domestic product per capita. Few studies assessed the affordability of CHW programs for government and/or partners. Evidence suggests that CHWs are often more cost-effective than alternative service or delivery modalities, particularly for child health. The evidence is however constrained by the heterogeneity of methods and reporting standards. To best guide future implementation of CHW programs, future research should focus on whether these interventions are affordable to governments and/or partners.

## Introduction

A disproportionate number of maternal, newborn, and child deaths globally occur in low- and middle-income countries (LMICs) [1]. Two regions in particular - sub-Saharan Africa and South Asia - accounted for more than 80% of the 4.9 million under-5 deaths in 2022 [2]. Reproductive, maternal, newborn and child health (RMNCH) complications are more prevalent in these regions due to a multitude of factors, including limited access to healthcare services, inadequate maternal and child nutrition resources, insufficiently skilled birth attendants, poor sanitation, and higher rates of infectious diseases [3]. Socioeconomic challenges and weak health systems further hinder access to essential health services, exacerbating the vulnerability of mothers, newborns and children in LMICs, leading to higher mortality rates [3].

One way to address some of these challenges, especially access to community-based care, is through the deployment of Community Health Workers (CHWs) [4]. The exact definition of CHWs varies between countries [5], however, they are broadly defined as individuals who undergo basic healthcare training and provide essential health services, education, and support directly to individuals in their communities [6,7]. CHWs serve as a vital link between communities and the formal health system, bringing health services closer to the people, including through performing tasks traditionally handled by doctors and nurses in areas that are physically or socially isolated from the formal healthcare system [7]. They are uniquely positioned to deliver healthcare services because of their integration within communities and often share the same cultural background as those they serve [7]. In the context of reproductive and MNCH (RMNCH), CHWs can provide essential maternal care services, including but not limited to, antenatal care home visits, birthing assistance, postpartum home visits, and child care services such as assessments of newborns and Integrated Community Case Management (iCCM) - a strategy to improve access to essential treatment services for children [7,8].

There is evidence for the role of CHWs in providing high-quality RMNCH [9], as well as a widespread perception that CHWs are “cheap” compared to other cadres of health workers [10]. However, to support broader implementation, there is a need for an updated, systematic and comprehensive evaluation of the costs, cost-effectiveness and affordability of CHW-led RMNCH programs [7]. Given limited public funds, this type of evidence is needed to guide the optimal allocation of scarce resources as well as the most efficient delivery of healthcare services [11]. The most recent study to broadly review the costs and consequences of CHW programs in LMICs was a scoping review by Vaughan *et al*., (2015) [12]. In this review, 36 economic evaluations of various CHW programs were identified. Vaughan et al., (2015) concluded that CHWs may be a cost-effective approach in some settings. However, this review did not focus specifically on RMNCH - only 13 of the studies within this original study related to RMNCH, nor did it assess the affordability of such programs.

Our review aims to provide an updated overview of the evidence on the costs, cost-effectiveness and affordability of CHW programs specific to RMNCH in LMICs between 2015 and 2024. Additionally, it assesses the methodologies used in these economic evaluations and examines reporting of costs, cost-effectiveness and affordability, to identify best practices and areas for improvement. In this article, we focus on two overarching areas of RMNCH: 1) Reproductive, maternal and newborn health, with sub-areas including: reproductive health, maternal and newborn care; and 2) Child health, with sub-areas including preventing child mortality and promoting survival, child development, nutrition, and infectious diseases.

## Methods

### Nature of review

Our scoping review forms part of an initial wider scoping review, conducted to identify and map the available evidence on economic evaluations of both vertical and integrated horizontal CHW programmes in LMICs published between 2015 and 2024. The protocol was uploaded to the Open Science Framework (OSF) on July 27, 2023 [13]. Due to the large number of studies identified and the heterogeneity between studies, the reporting of results has been divided into several publications, by health/disease area or type of CHW, for clarity and to facilitate comparisons between similar studies. This paper focuses exclusively on RMNCH.

A scoping review was chosen given the broad and varied nature of the field, with the goal of identifying updated evidence, mapping research methodologies, and highlighting knowledge gaps. This study was conducted according to the Preferred Reporting Items for Systematic Reviews and Meta-Analyses Extension for Scoping Reviews (PRISMA-ScR) guidelines [14]. The PRISMA-ScR Checklist is available in the Appendix (S1 File).

### Search strategy and study selection criteria

An initial search covering January 1, 2015, to July 11, 2023, was conducted in the following databases: Ovid MEDLINE(R) and Epub Ahead of Print, In-Process, In-Data-Review & Other Non-Indexed Citations and Daily (1946 to July 06, 2023); Ovid Embase Classic+Embase (1947–2023 July 07); Ovid APA PsycInfo (1806 to July Week 1 2023); Ovid Global Health (1910–2023 Week 26); Ovid AMED (Allied and Complementary Medicine) (1985 to June 2023); Cochrane Central Register of Controlled Trials (CENTRAL); Cumulative Index to Nursing and Allied Health Literature (CINAHL); Web of Science Core Collection; Scopus; and Latin American and Caribbean Health Sciences Literature (LILACS). To ensure this review was up-to-date, the search was re-run to capture relevant literature up to and including July 16, 2024.

Additionally, we searched the following sources to identify any relevant grey literature, using the search strategy outlined below and in supplementary files: Google Scholar, Bielefeld Academic Search Engine (BASE), DART-Europe E-theses Portal; e-theses online service (EThOS), Open Access Theses and Dissertations, and The OAIster database, plus websites of key organisations involved with CHWs (e.g., CHW Central, Community Health Impact Coalition, and Healthcare Information for All (HIFA.org)). Grey literature included (but was not necessarily limited to) theses or dissertations, preprints or unpublished research, and internal reports and the date span was January 1, 2015, to July 16, 2024.

The search strategy included all appropriate controlled vocabulary and keywords for ‘Community Health Workers’, ‘Economic Evaluations’ and ‘LMICs’, which are defined below. Reference lists of included studies were reviewed to identify any additional studies missed by database searches. Full database search strategies are available in the Supplementary Material (S1 Text).

### Community Health Workers

For this review, we drew upon previous literature [5,15,16] to define CHWs as healthcare workers who:

(a) are primarily based in the community providing primary healthcare services;
(b) are part of the health system (i.e., government or non-governmental organization supported CHWs), performing tasks related to health-care delivery, and/or health education, promotion, or care coordination; and
(c) have received organised training and/or certification, but do not have a tertiary-level degree such as a nursing or midwifery degree.

### Economic evaluations

Both full and partial economic evaluations were included. Full economic evaluations, as defined by Drummond et al., (2015) [17], compare the costs and outcomes of health interventions against alternatives such as the current standard of care or a no-intervention scenario. These may include Cost-Effectiveness Analysis (CEA), Cost-Utility Analysis (CUA), Cost-Benefit Analysis (CBA), Cost-Minimization Analysis (CMA), Cost-Consequence Analysis (CCA), Social Return on Investment (SROI), Multi-Criteria Decision Analysis (MCDA), Budget Impact Analysis (BIA), and Programme Budgeting and Marginal Analysis (PBMA).

Partial economic evaluations, on the other hand, consider costs and/or consequences without necessarily comparing alternatives or linking costs to benefits. They can include outcome descriptions, cost descriptions, cost-outcome descriptions, effectiveness evaluations, or cost analyses.

While full economic evaluations are preferred for guideline and policy development due to their comprehensive nature [11], partial economic evaluations are valuable for initial program development and in contexts where full evaluations are too costly, particularly in LMICs [18].

### LMICs

The World Bank classification of economies was used to categorise LMIC countries as either ‘low’, ‘lower-middle’ or ‘upper-middle’ income based on the costing date for each respective study [19].

### Reproductive, maternal and newborn health

In this review, we define reproductive health, maternal and newborn care as follows. Services focused on the health of women before and during pregnancy, childbirth, and the postpartum period (up to six weeks after delivery) could include, but are not limited to: Antenatal care (e.g., routine check-ups, nutrition counseling, screening for complications); Intrapartum care (e.g., skilled birth attendance, emergency obstetric care); Postnatal care (e.g., postpartum check-ups, family planning counseling, management of postpartum complications); Preconception care (e.g., nutritional support, folic acid supplementation, screening for infectious diseases) and Health education and counseling (e.g., birth preparedness, breastfeeding support, mental health support). Services focused on newborns (from birth through the first 28 days of life) could include but are not limited to: Essential newborn care (e.g., neonatal resuscitation, thermal care, early initiation of breastfeeding); Screening and management of neonatal conditions (e.g., neonatal sepsis, low birth weight); Immunization (e.g., polio, hepatitis B); Postnatal follow-up (e.g., growth monitoring, screening for developmental anomalies). Within this broad area of Reproductive health, maternal and newborn care, we distinguish two sub-areas: 1) Reproductive health, and 2) Maternal and newborn care.

### Child health

Child health extends beyond the neonatal period up to childhood - commonly up to five years of age. Child health services could include but are not limited to: Integrated Community Case Management (iCCM) (e.g., diagnosis and treatment of common childhood diseases such as pneumonia, diarrhea, and malaria); Nutritional support and supplementation (e.g., vitamin A, iron supplementation, therapeutic feeding for malnutrition); Routine immunizations (e.g., measles, diphtheria-tetanus-pertussis [DTP], Haemophilus influenzae type b [Hib]); Growth monitoring and health promotion (e.g., regular weight and height measurements, health and hygiene education); and Early childhood development interventions (e.g., psychosocial stimulation, early learning support). In this article, we focus on four sub-areas of Child health: 1) preventing child mortality and promoting survival, 2) child development, 3) nutrition, and 4) infectious diseases.

### Inclusion and exclusion criteria

Studies were included if they:

- Primarily evaluated CHW programs, excluding those focused exclusively on other healthcare professionals such as doctors, nurses, or midwives.
- Evaluated CHW programs focused on RMNCH.
- Provided details of an economic evaluation, including either full or partial evaluations.
- Were published between August 2015 and July 2024, as the previous review on this topic covered literature up to July 2015.
- Evaluated interventions or programs located in LMICs per the World Bank classification in the year the study was costed.

Studies were excluded if they:

- Were letters to the editor, commentaries, protocols, opinion pieces, policy briefings, or conference abstracts. Although systematic reviews were excluded, their reference lists were searched for potentially eligible studies.
- Assessed the economic impact of digital add-ons to CHW programs (e.g., mobile phone interventions), as the focus of our review was on the economic evaluation of CHW-led interventions themselves, not digital add-ons.

No restrictions were placed on the time frame of the analysis or language of publication. Although the search was conducted in English, full texts were reviewed in any language. Studies were not excluded based on quality due to the high diversity in study types and the interest in exploring the breadth of available evidence. Full eligibility criteria are detailed in the PICO framework in the Supplementary Material (S2 Text).

### Study screening process

Following a search of the databases and grey literature by a qualified information search specialist, citations were exported to the Covidence platform [20]. Duplicate results were removed using an automated ‘de-duplicate’ feature within Covidence. A team of 18 researchers took part in the screening process. Each title and abstract were reviewed independently by two reviewers. Conflicts were resolved by a third reviewer. Two independent reviewers conducted a full-text review to determine final inclusion or exclusion. Conflicts at this stage were resolved by a third reviewer.

### Data extraction

Data were extracted into a custom Google Sheets document and rigorous quality control was conducted on the extractions. A third reviewer (a health economist) was available to resolve any disagreements. The data extraction form was tested for user-friendliness and completeness by all extractors independently and discussed during a joint video conference call. The spreadsheet captured the article meta-data, information about the study site and CHWs involved in the study, methodological and reporting data, as well as outcomes and cost data.

Outcomes were categorised into five categories: (i) Service Provision (e.g., visits, number of medications distributed, number of household visits); (ii) Population Coverage (e.g., households covered); (iii) Mortality and Morbidity outcomes (e.g., reduction in mortality, lives saved); (iv) Cost Savings and Cost Recovery outcomes (e.g., amount of money saved); and (v) Societal Outcomes (e.g., economic growth).

We documented whether costs were reported in the following categories: (i) Cost per CHW; (ii) Cost per Consultation; (iii) Cost per Service; (iv) Cost per Capita; or (v) Cost per Beneficiary. We also extracted other cost reporting that is specific to RMNCH such as the Cost per Mother-child dyad seen. We documented whether cost per outcome was reported in the study (i.e., Cost per Disability-Adjusted Life Year (DALY) Averted). All costs were converted to and reported in 2024 US$ to facilitate comparison. For costs reported in US$, we first converted costs to local currency units (LCUs) of the same year using that year’s exchange rate (World Bank, ‘US$ per LCU, period average’). With the costs reported in US$ now in LCUs, and for costs originally reported in LCUs by the resource, we inflated costs to 2024 LCUs using LCU inflation rates reported by the International Monetary Fund (‘inflation, average consumer prices’). With all costs in 2024 LCUs, we converted costs to 2024 US$ using the ‘LCU per US$, period average’ official exchange rate for 2024. For cost-effectiveness, we report the incremental cost-effectiveness ratios (ICERs) converted to 2024 US$. An ICER reports the difference in total costs (incremental cost) of the CHW program and the comparator divided by the difference in the chosen measure of health outcome or effect (incremental effect) to provide a ratio of ‘extra cost per extra unit of health effect’.

We also report on whether the study authors drew conclusions on the cost-effectiveness and affordability of the CHW program. To determine cost-effectiveness, we looked for comparisons against: (i) thresholds (willingness to pay or gross domestic product (GDP)/capita); or (ii) an alternative service or delivery modality, such as facility-based care. For affordability, we noted whether authors reported how the intervention affects the overall healthcare budget (budget impact analysis), including whether the intervention is affordable within the current budget constraints. That said, we report on cost-effectiveness and affordability based on the authors’ determination or conclusions from the original study, regardless of whether a threshold was used.

We used Microsoft Excel to organise and analyse extracted data.

### Patient and public involvement

Patients and the public were not consulted as part of this scoping review.

### Ethics approval

A self-assessment was conducted via the University of Washington Human Subjects Institutional Review Board (IRB) which determined that this study was not human subjects research and did not require IRB review.

## Results

### Search results

The initial broader literature search, which included maternal, newborn and child health, and other health areas (e.g., (i) non-communicable diseases, (ii) neglected tropical diseases, (iii) infectious diseases [21], and (iv) horizontal, integrated CHW programs [22] - *some of which are currently under review or in press*), yielded 9,790 articles. This was reduced to 5,663 after the removal of duplicates. A further 5,345 studies were excluded following abstract screening and an additional 170 after full-text review.

**Fig 1:**
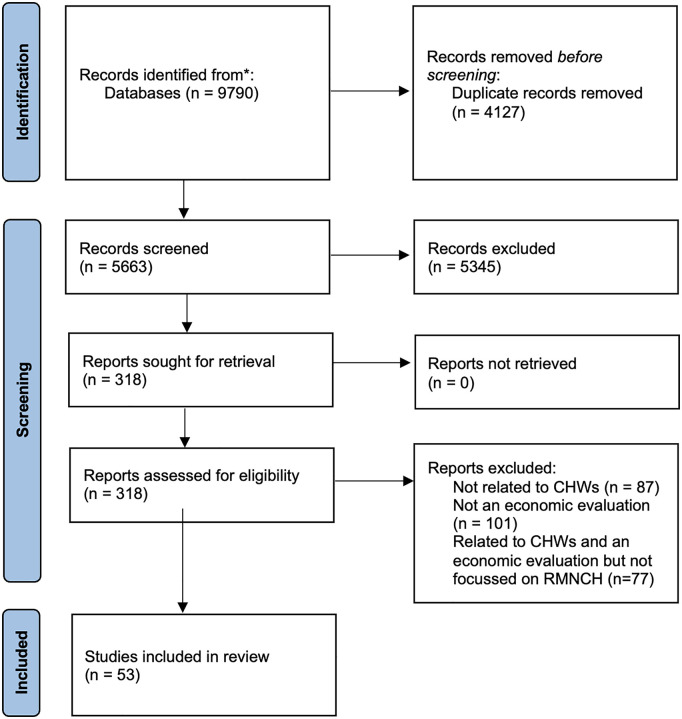
PRISMA-ScR flow diagram. A total of 9,790 records were identified through database searches. After removing 4,127 duplicates, 5,663 records were screened. Of 318 full-text reports assessed for eligibility, 53 studies met inclusion criteria. Exclusions were based on relevance to CHWs, presence of economic evaluation, and RMNCH focus.

After coding studies by health/disease area, this process resulted in 53 RMNCH studies being included in this review. Each of the 53 studies contained multiple scenarios (for example data reported from various countries, various intervention models, or varying study perspectives) which are described in the findings tables. Further details can be found in the PRISMA flow chart (Fig 1).

This review includes several articles that report summarized data from individual studies, which are also part of our review [23–30]. These summary papers sometimes took the form of a meta-analysis, and sometimes simply re-analyzed the original data differently, producing different results. To avoid duplicating cost data across these summary papers, we conducted a comprehensive screening of each situation where both summarized data and an individual article reporting on the same study were included. The extracted data from these papers were exported and compared row by row in an Excel spreadsheet, a process carried out by three of the authors. After a thorough comparison, we found no overlap in the reported cost data between the summary and individual papers, and as a result, we included all of them in this review.

The following two subsections present summarized findings for cost, cost-effectiveness and affordability findings separately for (1) RMNH (2) Child health. Each section describes the CHW programs and alternatives assessed and reports on the relevant cost, cost-effectiveness and affordability findings. For more detailed findings, please see the supplementary files (S1 Data and S2 Data).

1 **Reproductive health, maternal and newborn care**

We found a total of 21 reproductive, maternal and newborn health-focused studies, covering 77 scenarios across reproductive health (n = 4 studies, n = 5 scenarios) [31–34] and maternal and newborn care (n = 17 studies, n = 72 scenarios) [23–29,35–44]. These were conducted in 12 countries: Bolivia [24,25], Ethiopia [24–26,32], Ghana [24,25,27], India [35,41,42], Kenya [34], Malawi [24,25,39], Mozambique [35], Pakistan [35,40,43], Sierra Leone [37], South Africa [24,25,36,38], Tanzania [24,25,28,31], and Uganda [24,25,29,33]. CHWs were mostly compared to unspecified “standard” care. Table 1 summarizes the findings from the reproductive health, maternal and newborn care studies.

**Table 1:** Reproductive, maternal and newborn health findings.

Topic	Reproductive health	Maternal and newborn health
**Number of studies**	4	17
**Number of scenarios**	5	72
**Number of countries**	4 (Ethiopia, Kenya, Tanzania, Uganda)	11 (Bolivia, Ethiopia, Ghana, India, Malawi, Mozambique, Pakistan, Sierra Leone, South Africa, Tanzania, Uganda)
**Summary of CHW tasks and responsibilities**	All scenarios included aspects of CHWs conducting home visits (n = 5), with four of those providing contraceptives (n = 4) and one focused on broader maternal health prevention and promotion (n = 1).	All scenarios included aspects of CHWs conducting home visits (n = 72), during which they provided counseling and care during and after pregnancy to improve newborn outcomes.
**Summary of comparators**	Two scenarios compared community-based and facility-based distribution of two contraception options, while one compared outcomes to other countries. Two scenarios did not include comparators (n = 2).	Just under half of scenarios (n = 33) compared the CHW intervention to unspecified standard care. In five scenarios the CHW intervention was compared to various scaled-up models of the same intervention. The remaining scenarios (n = 32) did not include a comparator.
**CHW compensation method (2024 US$)**	CHWs were volunteers (n = 2) or received incentives in the form of backpacks, mobile phones, SMS fees and internet access (n = 2). The remaining CHWs were remunerated through microloans (n = 1).	In 23 scenarios, CHWs were salaried; in 17 scenarios, CHWs received stipends; and in 7 scenarios, CHWs received incentives, including bicycles (n = 5) and no-interest loans (n = 1), with one scenario not disclosing the incentive. In four scenarios, CHWs received compensation for attending training and meetings. In 17 scenarios, CHWs were volunteers, while the remaining scenarios did not document remuneration (n = 4).
**Cost per beneficiary (2024 US$)**	$45.51 (n = 1)	$0.19 to $1,547 (n = 57)
Other cost outcomes***	Cost per service $9.61 to $9.64 (n = 2)	Cost per consultation $0.45 to $52.91 (n = 53) Cost per capita per year $0.09 to $20.25 (n = 41) Cost per CHW per year $21 to $732 (n = 9) Cost per DALY averted $64 to $478 (n = 3)
**Cost-effectiveness criteria and conclusions**	The majority of scenarios were deemed cost-effective when compared to an alternative (n = 2) or willingness to pay threshold (n = 1, total n = 3). The remaining scenarios did not assess cost-effectiveness (n = 2).	Of the 48 scenarios that examined cost-effectiveness, the majority were deemed cost-effective (n = 37), based on GDP per capita (n = 29), comparison with alternatives (n = 6), return on investment (n = 1), and willingness to pay (n = 1). In four scenarios CHWs were “likely” cost-effective based on willingness to pay (n = 3) and GDP per capita (n = 1). Three scenarios concluded the CHW intervention was not cost-effective as implemented according to willingness to pay and GDP per capita thresholds, but had a “high chance” of being cost-effective if the number of contacts between CHWs and pregnant women increased. Two scenarios were not cost-effective according to GDP per capita and comparison with alternatives. Two scenarios reached unclear conclusions with no threshold specified. The remaining scenarios did not assess cost-effectiveness (n = 24).
**Affordability conclusions**	None of the scenarios assessed affordability (n = 5).	The majority of scenarios did not assess affordability (n = 39). Of those that did assess affordability, most did not reach a conclusion (n = 29), while the remaining scenarios found CHWs to be affordable based on public health expenditure per capita (n = 3).

#### Remuneration.

Nearly a third of scenarios (n = 23) [24,25,35,39,44,45] included salaried CHWs, with monthly salaries ranging from $146 to $311 in low-income countries and reaching $3,216 in South Africa, an upper-middle income country. In 17 scenarios [25,27,28,35,38,41,42], CHWs received a monthly stipend, which ranged from $3.44 in Ghana to $277 in South Africa. In 13 scenarios [25,26,28,43], CHWs were non-remunerated volunteers. In the remaining scenarios, CHWs received in-kind incentives (n = 9) [31,34,37,39], microloans (n = 1) [33], were valued using hypothetical salaries (n = 6) [29,33] or no other information was provided (n = 4) [24,40].

#### Cost metrics.

Across all 77 scenarios, the most commonly reported cost metric was cost per beneficiary per year (n = 58) [23,25–29,32,35,36,38,39,41,43], which ranged from $0.19 to $1,547. Additional reported cost metrics included cost per capita per year (n = 41) [23,25–29,36,37,39] ranging from $0.09 to $20.25, cost per consultation (n = 53) [23,25–29,35,36,39] ranging from $0.45 to $52.91, cost per CHW per year (n = 9) [23,24,28] ranging from $21 to $732, cost per DALY averted (n = 3) [26,40] ranging from $64 to $478 and cost per service (n = 2) [33] ranging from $9.61 to $9.64.

#### Cost-effectiveness.

Cost-effectiveness was assessed for 51 scenarios, with the majority (40/51) [25–28,33,34,37,40–44] concluding CHWs were cost-effective, with another 4 scenarios [39] concluding that the CHW intervention was “likely” cost-effective. This conclusion was based on GDP per capita (29/40) [25–27,37,40–42] or willingness to pay (2/40) [34,43] thresholds, comparing CHWs with alternatives (8/40) [28,33] and return on investment results (1/40) [44]. Examples of cost-effective CHW interventions included the distribution of contraceptives (compared to facility-based distribution and self-injections) in Burkina Faso, Uganda and Senegal [33], CHW-led interventions for newborn health through home visiting (compared to standard facility-based care) in Ethiopia and Pakistan [26,40], and an intervention to prevent and treat perinatal depression (compared to standard facility based care) in Pakistan [43]. For two scenarios, the conclusion was that the CHW intervention was not cost-effective, based on the cost per life year saved far exceeding both the GDP per capita threshold (n = 1) [37] and the cost per life year saved of other exclusive breastfeeding interventions implemented in the same context though evaluated in other studies (n = 1) [38]. Three scenarios found the CHW intervention as implemented not to be cost-effective, but predicted it would be cost-effective if the number of contacts between CHWs and pregnant women increased [35].

Less than half of scenarios (n = 32) [23,25,26,29,36,39] explicitly considered the affordability of CHWs, and only three of these [23,29] concluded CHWs to be affordable based on program costs as a share of public health expenditure per capita (0.63% to 1.8%). These affordable scenarios involved CHWs conducting pregnancy visits, delivering Integrated Management of Childhood Illness, and occasionally attending home deliveries across varying coverage levels in Bolivia. The remaining scenarios (n = 29) had no affordability conclusions [25,26,36,39].

2 **Child health**

We found a total of 32 child health studies covering 84 scenarios across preventing child mortality and promoting survival (n = 9 scenarios) [46–49], childhood development (n = 7) [50–52], nutrition (n = 30) [53–65], and child-specific infectious disease prevention and management (n = 38) [30,66–76]. These were conducted in 18 countries: Bangladesh [53], Burundi [72], Ethiopia [30,48,73], Ghana [30,46,67], India [57,66], Kenya [50,56,65,71], Madagascar [74], Malawi [32,50], Mali [30,48,59,62], Mozambique [30], Nepal [64], Niger [30,49,54,63], Nigeria [55], Pakistan [58], Rwanda [52], South Africa [47,51], Tanzania [61] and Uganda [60,66–70,75,76]. CHWs were mostly compared to alternatives such as facility-based services, unspecified “standard” care or drug sellers (trained or untrained workers at private drug shops). Table 2 summarizes the findings from the child health studies.

**Table 2:** Child health findings.

Topic	Preventing child mortality and promoting survival	Childhood development	Nutrition	Child-specific infectious disease prevention and management
**Number of studies**	4	3	13	12
**Number of scenarios**	9	7	30	38
**Number of countries**	6 (Ethiopia, Ghana, Malawi, Mali, Niger, South Africa)	3 (Kenya, Rwanda, South Africa)	10 (Bangladesh, India, Kenya, Mali, Nepal, Niger, Nigeria, Pakistan, Tanzania, Uganda)	11 (Burundi, Ethiopia, Ghana, India, Kenya, Madagascar, Malawi, Mali, Mozambique, Niger, Uganda)
**Summary of CHW tasks and responsibilities**	The majority of scenarios reflected CHWs conducting home visits (n = 7). Other CHW tasks included reporting on pregnancies, births, and deaths (n = 2).	All scenarios included aspects of CHWs conducting home visits (n = 7), with five scenarios focused on child interventions and two on caregiver interventions.	The majority of scenarios reflected CHWs conducting screening and treating for malnutrition (n = 18). In some scenarios, CHWs also distributed micronutrients (n = 7) or provided breastfeeding support (n = 1). Four scenarios presented various management strategies of a nutrition program, such as MoH take-over and NGO management.	The majority of scenarios included integrated community case management (iCCM) specifically (n = 23) or other community-based management of diseases (n = 14, total n = 37). One included CHWs providing measles vaccinations.
**Summary of comparators**	The majority of scenarios compared various channels for delivery of interventions, such as door-to-door vs. fixed points. (n = 6). Three scenarios had no comparator (n = 3).	Four scenarios compared different delivery modalities, including the use of Mentor Mothers, group sessions and mixed methods. The remaining scenarios compared various forms of cash-for-work schemes that CHWs helped administer, including a basic scheme of cash-for-work, and expanded schemes including financial literacy and asset transfers (n = 4).	CHWs were most commonly compared against health facility delivery (n = 11). Also common was intervention vs unspecified standard care (n = 7) or CHWs vs mothers (n = 1). Some scenarios compared varying CHW supervision strategies (n = 6) or different districts/agroecological zones (n = 4). One had no comparator.	The most common comparators were standard care at health facilities (n = 16) or different distribution methods for drugs (n = 9). Four scenarios compared different coverage levels of community-based treatment of childhood pneumonia, while one scenario compared CHWs against other community health activities (n = 1). One scenario compared the opportunity cost of CHWs’ time against the lowest wage rate in the country’s health system. Two scenarios had no comparator.
**CHW compensation method (2024 US$)**	In most scenarios, CHWs received a stipend (n = 6). In others, CHWs were salaried (n = 2) or received an incentive (airtime) (n = 1).	CHWs were both salaried (n = 2) or received a stipend (n = 2). For the remaining scenarios, the remuneration model was not documented (n = 3).	The majority of scenarios reflected salaried CHWs (n = 14). Some CHWs received a stipend (n = 8) or were valued using a hypothetical stipend (n = 4). In four scenarios CHWs were not compensated.	In 12 scenarios, CHWs received a non-monetary incentive package featuring items to use in their work, such as bicycles, rainboots, t-shirts, etc. In 11 scenarios, CHWs were uncompensated. In other scenarios, CHWs received a salary (n = 7) or a stipend (n = 2). CHW remuneration was not documented in the remaining scenarios (n = 6).
**Cost per beneficiary (2024 US$)**	$3.07 to $147.15 (n = 2)	$110.13 to $482.45 (n = 7)	$3.17 to $380.18 (n = 27)	$0.02 to $123.48 (n = 22)
Other cost outcomes***	Cost per capita per year $0.40 to $6.14 (n = 5)	Cost per consultation $17.99 to $40.20 (n = 3)	Cost per child treated $176 to $380 (n = 11) Cost per child recovered $194 to $380 (n = 8) Cost per death averted $1,890 to $15,116 (n = 7) Cost per DALY averted $28 to $400 (n = 5)	Cost per capita per year $0.12 to $4.05 (n = 10) Total cost per CHW $142 to $2,464 (n = 7) Cost per DALY averted $9 to $206 (n = 7)
**Cost-effectiveness criteria and conclusions**	The majority of scenarios did not assess cost-effectiveness (n = 8). One scenario reported that CHWs were likely to be cost-effective given WHO thresholds linked to GDP per capita and positive cost/benefit results.	In two scenarios, CHWs were deemed to be cost-effective, based on the cost-benefit ratio and return on investment (n = 2). One additional scenario (the expanded cash-for-work scheme integrated into government) was more cost-effective than the two alternatives: the basic scheme and the expanded scheme not integrated into government. Two scenarios did not assess cost-effectiveness (n = 2).	The majority of scenarios concluded that CHWs were cost-effective, compared with alternatives (n = 14) and GDP per capita (n = 3, total n = 17). Two scenarios found CHWs to be not cost-effective when compared with mothers screening for severe acute malnutrition (SAM) or treatment and counselling for SAM delivered via outpatient facility-based care. Eleven scenarios did not assess cost-effectiveness (n = 11).	The majority of scenarios were cost-effective when compared with alternatives (n = 7), GDP per capita (n = 6) and willingness to pay (n = 3, total n = 16). Three scenarios had mixed results: CHWs were more cost-effective than the “do nothing” alternative, but less cost-effective than drug sellers (n = 3). Three scenarios found CHWs selling diarrhea treatment or vouchers to be less cost-effective than free distribution. One scenario had unclear findings since a willingness to pay threshold did not exist in that setting. Fourteen scenarios did not assess cost-effectiveness (n = 14).
**Affordability conclusions**	Most scenarios did not assess affordability (n = 7). One scenario concluded CHWs would be “affordable”, based on their impact on the health budget (n = 1) and one scenario was inconclusive due to the lack of a rigorous analysis of national budget constraints (n = 1).	The majority of scenarios did not assess affordability (n = 5). Two scenarios were both “cost-saving” and affordable, based on expected health system costs associated with low birth weight and undernutrition (n = 2).	The majority of scenarios did not assess affordability (n = 23). Of those that did assess affordability, six reached no conclusion (n = 6) while one scenario found the cost of CHWs to be “not insignificant” but still affordable (n = 1).	The majority of scenarios did not assess affordability (n = 31). Six scenarios were inconclusive on affordability according to public health expenditure per capita, which was estimated to range from 0.6% to 7.4% of public health expenditure per capita. The remaining scenario was not affordable according to willingness to pay due to insufficient public health expenditure.

#### Remuneration.

Nearly a third of scenarios (n = 25) reflected salaried CHWs [32,48,49,53,58,60–62,64,65,75], with monthly salaries ranging from $49 to $284 (low-income countries) and $312 in South Africa, an upper-middle income country. In 22 scenarios, CHWs received a stipend [30,48–50,54,55,61,64], which ranged from $46 to $268 per month in low-income countries and $20 to $150 in lower-middle-income countries. In 10 scenarios, CHWs were non-remunerated volunteers [30,53,57,65,66,71,72,74]. In the remaining scenarios, CHWs received in-kind incentives (n = 15) [48,67,70,76] or were valued using hypothetical wages (n = 5) [68,69], or information about their remuneration was not provided by study authors (n = 7) [52,75].

#### Cost metrics.

Across all 84 scenarios, the most commonly reported cost metric was cost per beneficiary (n = 58) [48–52,54,57–65,67,69,71,72,74–76], which ranged from $0.02 to $482. Additional reported cost metrics were cost per capita per year (n = 14) ranging from $0.12 to $4.05 [30,47,49,69], cost per service (n = 14, $6.06 to $367) [48,60,75], and cost per consultation (n = 5) from $0.26 to $40 [30,52]. Cost per DALY averted (n = 12) ranged from $9 to $400 [53,55,57,65,66,75], cost per child treated for SAM (n = 6) [58,59,61,62,65] from $176 to $380, cost per child recovered from severe acute malnutrition (SAM) (n = 6) from $194 to $380 [39,58,59,61,62,65], and cost per death averted (n = 6) from $1,890 to $15,116 [39,58,59,61,62,65]. The total cost per CHW (n = 6) ranged from $142 to $2,464 [30].

#### Cost-effectiveness.

Cost-effectiveness was assessed for 49 scenarios, with the majority (37/49) [46,50,52,53,55,57,59–61,63,65–67,71–73,75,76] concluding CHWs were cost-effective. This conclusion was reached when comparing CHWs with alternatives (22/37) [52,59–61,63,65,67,71] or cost-benefit ratio and return on investment results (2/37) [50], or based on GDP per capita or willingness to pay thresholds (13/37) [46,53,55,57,66,72,73,75,76]. Examples of cost-effective CHW interventions included group-based parenting interventions (compared to standard care) [50], community-based screening and treatment of malnutrition (compared to standard care at health facilities) [52,55,57,59,61,65], vaccinations (compared to standard care) [66], and iCCM (compared to standard care at health facilities) [71,72,76].

In the remaining 12 out of 49 scenarios, the use of CHWs was not concluded to be cost-effective. In two scenarios CHWs were not as cost-effective a non-CHW alternative: in rural Niger, mothers screening for malnutrition using mid-upper arm circumference achieved comparable coverage at a substantially lower cost, and in Sindh Province, Pakistan, outpatient facility-based treatment for uncomplicated severe acute malnutrition was found to be slightly more cost-effective than CHW-delivered care [54,58]. In three scenarios CHWs were less cost-effective than drug sellers (for iCCM for treatment of under-five febrile cases of malaria, pneumonia, diarrhoea) [75]. Six scenarios compared different CHW-delivered interventions, with some models found to be more cost-effective than others [52,75]. For example, in Uganda, CHWs delivering diarrhoea treatment through free home delivery represented the most cost-effective model, followed by home sales, while voucher distribution was less consistently cost-effective [75]. The conclusion of the remaining scenario was unclear due to the absence of a defined threshold [74]. Thirty-five scenarios did not assess cost-effectiveness [30,47–49,51,56,62,64,68,69,73].

Only 18 scenarios explicitly considered the affordability of CHWs [30,46,47,51,55,64,65,74], and only four of these concluded CHWs to be affordable [47,51,55]. Two of these scenarios noted that CHWs generated cost savings compared to nurses, both from South Africa, where employing CHWs to provide home visits, health screenings, immunizations, and support for mothers and children under six was more cost saving than nurse-led delivery [51]. The other two affordable scenarios compared the cost of community interventions to reduce child mortality and manage acute malnutrition against public health expenditures or budgets in South Africa and Northern Nigeria [47,55].

### Methodological findings across all studies

In this section, we summarise selected methods-related findings across all included studies (n = 53) and scenarios (n = 161).

Across studies in the two focus areas, the most commonly reported outcomes were cost per beneficiary (72% of scenarios), cost per consultation (36%) and cost per capita (34%). Nine percent of scenarios reported cost/DALY averted.

The majority (55%) of scenarios took a provider perspective, either explicitly stated (n = 67 scenarios) or inferred (n = 22). Fifty-three percent of scenarios employed a one-year time horizon, which is helpful for comparability across studies. Recurrent costs were included in 98% of scenarios. There was considerable variability in reporting on training costs (76% of scenarios), non-training capital items (meaning items used over one year, such as equipment) (76%), indirect costs or overheads (66%), and out-of-pocket and opportunity costs (56%). A limited number of studies reported costs averted (12%).

Only nine studies (representing 11% of scenarios) used the Consolidated Health Economic Evaluation Reporting Standards (CHEERS) checklist [12], despite it being the leading guidance for authors to follow to ensure that health economic evaluations are identifiable, interpretable, and useful for decision-making.

## Discussion

This scoping review provides an updated synthesis of the evidence on the costs, cost-effectiveness, and affordability of CHW programs focused on two broad areas of RMNCH interventions in LMICs: reproductive health, maternal and newborn care, and child health. Our findings are based on 53 studies and 161 scenarios published between 2015 and 2024, spanning sub-Saharan Africa, South Asia, and, to a lesser extent, Latin America and the Caribbean. Our comprehensive analysis of 100 scenarios across reproductive health, maternal and newborn care and child health domains overwhelmingly suggests that CHWs can be cost-effective interventions to support mortality reduction, child development, nutrition, and infectious disease management. However, we interpret these findings with caution given the differences across the two areas and by sub-area, the use of different criteria to judge cost-effectiveness, particularly in light of recent methodological changes in how cost-effectiveness is assessed and interpreted. Across all sub-areas, the depth and consistency of economic evidence remain limited, largely due to the heterogeneity in both intervention and methods in the included studies. Furthermore, cost ranges may be too wide to be useful for planning and budgeting purposes, and there is a lack of affordability analyses.

### Cost findings and variability

Across reproductive health, maternal and newborn care and child health, CHW-led interventions in our review were associated with widely varying cost estimates, as reflected in common metrics such as cost per beneficiary (ranging from $0.02 to $1,547), cost per capita (ranging from $0.09 to $20.25), and cost per consultation (ranging from $0.26 to $52.91). The wide ranges reflect substantial differences in program scale, intensity, local resource costs, and the scope of CHW responsibilities. For example, CHWs in the included studies performed a variety of tasks—from providing basic home-based care and health education to screening for complications and referring patients for facility-based care—complicating attempts to derive standardized “typical” costs. There are significant contextual differences in CHW employment, integration, and support across countries. Moreover, compensation strategies for CHWs varied from salaries (with amounts as high as $3,216 per month in some maternal care scenarios) to stipends of only a few dollars per day, and in some cases, non-remunerated volunteer labor. These differing remuneration models do not always align with global policy recommendations to formally compensate CHWs and make cross-study comparisons challenging [77]. Finally, there are also methodological differences between studies which may be driving cost findings, such as the inclusion of different cost items or the use of different time horizons. Together, these issues make it challenging to compare cost findings across studies and deem it unlikely that cost findings from this review are useful for planning and budgeting purposes at sub-national, national or global levels. We caution against overgeneralizing findings without considering the broader structural social and health determinants that drive maternal and child health outcomes.

### Cost-effectiveness

This review documents 100 scenarios where authors assessed cost-effectiveness, over three-quarters of which found CHWs to be cost-effective compared to different alternatives or thresholds. Within child health, the evidence indicates that CHW interventions are cost-effective, based on favorable findings in 37 of 49 scenarios where cost-effectiveness was assessed. In 22 of 37 scenarios, CHWs were found to be more cost-effective than alternatives such as health facility-based service delivery. Examples of cost effective child health interventions included parenting groups, screening and treatment of malnutrition, and iCCM. For maternal health related interventions, examples of cost effective interventions included distribution of contraceptives and newborn care through home visiting. This does not necessarily mean they were less expensive than alternative care: cost-effectiveness considers both costs and effectiveness, and in some cases CHWs were more expensive than alternative delivery mechanisms but also more effective, driving their cost-effectiveness. Other scenarios found CHWs to be cost-effective when compared against thresholds such as GDP/capita or willingness to pay. Although our scoping review did not include assessing the quality or robustness of authors’ reported conclusions about cost-effectiveness, we do find it important to note that the GDP/capita threshold in particular is no longer widely accepted for concluding about cost-effectiveness. Multiple critiques have noted that it fails to consider local resource constraints and results in too many interventions being considered cost-effective, meaning findings cannot be useful for supporting decision-making. It is possible that CHWs in these scenarios would still be found to be cost-effective if findings were considered against new, alternative thresholds that are lower than those based on GDP/capita, but this analysis was outside the scope of our review. Nevertheless, the predominance of “cost-effective” findings based on comparison with an alternative does reinforce the general promise of child health interventions delivered by CHWs, especially in settings where resource constraints limit access to facility-based care and where CHWs can help overcome barriers such as travel distance and shortage of skilled providers.

There are some differences by sub-area. Within child health, the evidence around CHWs involved in nutrition and child-specific infectious disease prevention and management is sizeable (n = 68) and overall positive about the cost-effectiveness of CHWs, while the evidence base is much smaller (n = 16) and less conclusive around interventions on preventing child mortality or promoting child survival as well as child development.

For reproductive health, maternal and newborn care, while the majority of scenarios where authors assessed cost-effectiveness were reported to be cost-effective or likely cost-effective (44 of 51 scenarios), a large number of these are based on the GDP/capita threshold, subject to the limitations mentioned above.

Amongst all 81 scenarios concluding CHWs were cost-effective or likely cost-effective, CHWs received a salary or stipend or were valued hypothetically in around half of these, with other scenarios representing unpaid (volunteer) CHWs or situations where CHWs received non-monetary incentives such as raincoats. This brings into question whether the cost-effectiveness evidence would still be positive in the remaining scenarios if the CHWs were remunerated in alignment with global policy recommendations.

### Affordability and budget impact

A program can be cost-effective yet still remain unaffordable for governments if the total budget required exceeds available financial resources. Thus, an affordability or budget impact assessment - and not just cost-effectiveness results - are critical for translating evidence into policy. Despite the positive cost-effectiveness findings, only 18 child health scenarios examined affordability or budget impact. For reproductive health, maternal and newborn care, only 29 scenarios examined affordability. Importantly, only two child health scenarios (and no reproductive health, maternal and newborn care scenarios) concluded that CHWs would be affordable given that they generated cost savings compared to alternative cadre (nurses), while five concluded that program costs as a percentage of public health expenditure per capita would be considered affordable; only two of the five reported this percentage (0.6% and 1.8%). Other studies reported the percentage without drawing a conclusion about the actual affordability of the reported percentage (0.3% to 7.4%). As with the cost-effectiveness results, the type and amount of CHW remuneration are crucial here. Further, when CHWs are paid, these workforce costs can appear significant relative to tight government budgets, especially if savings generated as a result of task shifting from more expensive cadres is not considered. Overall we find too little evidence to draw conclusions about CHW affordability in LMICs.

### Implications for policy and practice

Taken collectively, these findings suggest that certain CHW-led RMNCH programs can be a cost-effective strategy to improve outcomes compared to alternative delivery modalities, such as health facility-based service delivery, particularly for child health. However, given the heterogeneity of interventions, study methods and study contexts, these findings should be interpreted cautiously. To facilitate evidence-informed policy decisions, future economic evaluations must adopt rigorous and transparent methodologies, explicitly address affordability, and use consistent, comparable outcome measures such as DALYs. Policymakers should interpret cost-effectiveness and affordability findings in conjunction with local budget realities and other decision-making criteria, including equity, population coverage, and the health system’s capacity to absorb and supervise CHWs at scale.

### Strengths and limitations of this review

A strength of this review is its comprehensive scope, including both published and gray literature across multiple regions and a wide range of RMNCH interventions. The systematic approach and disaggregation of 161 scenarios allow for a granular analysis of how RMNCH-focused CHW-led programs have been evaluated economically. However, the inherent limitations of a scoping review remain: we did not exclude studies based on quality (nor did we conduct a quality analysis) [78], and our synthesis is constrained by the heterogeneity of methods and reporting standards. Moreover, we did not attempt any reinterpretation of data reported using current cost-effectiveness thresholds, but instead reported them as the authors did in respective papers. Finally, while we have brought all cost-related findings to 2024 US$ for comparability, we have not adjusted for purchasing power parity.

### Reflexivity statement

We adhered to the consensus statement on equitable authorship in international research collaborations as outlined by Morton et al., (2021) [79]. The following reflexivity statement is provided in that context. This research was conducted by a multidisciplinary, global team comprising researchers and practitioners from LMICs where CHW programs are implemented, such as Malawi, Rwanda, Uganda, Kenya and Liberia. Team members hold positions in academic institutions, non-governmental organisations, and frontline health services (and included CHWs), enabling us to integrate both theoretical and practical insights into our study. All members who contributed to the study design, implementation, analysis, and writing of this paper have been included as co-authors. We acknowledge that the authorship team does not include LMIC government stakeholders, who are a key audience for this research. While many team members have extensive experience engaging with government health systems, this absence may limit study findings. That said, this study was undertaken by the Community Health Impact Coalition (CHIC), a collective of thousands of CHWs and dozens of global health organisations spanning over 60 countries in five WHO regions. The research questions, data collection methods, and analysis were shaped by CHIC’s commitment to understanding the drivers of impact and quality in CHW-delivered care globally.

Importantly, this work was also shared with CHWs to explore their opinions and solicit their feedback. Their insights were integral to refining our approach and ensuring the relevance of our findings to those most directly impacted by CHW programs. For a detailed reflexivity checklist, please refer to the Supplementary Material (S2 File).

### Future research

Several key areas warrant further exploration. First, consistent use of a standardized reference case - specifying the analytic perspective, time horizon, and outcome measures - would greatly enhance comparability. Second, studies should incorporate budget impact analyses to provide more rigorous assessments of affordability, addressing a critical gap faced by policymakers in determining whether and how to scale CHW programs. As health service delivery shifts to integrated models, future research is likely to consider more horizontal, integrated CHW programs as opposed to the vertical ones assessed in this review.

## Conclusion

The evidence base on CHW involvement in RMNCH has expanded significantly since the Vaughan et al. (2015) review. On the whole, the currently available evidence allows us to conclude that CHWs are often more cost-effective than similar care delivered by other health professionals, in line with previous findings from the earlier, smaller evidence base. Nevertheless, there is too little evidence to conclude whether these interventions are affordable to governments, an important area for future research.

## Supporting information

S1 Data Detailed findings, tables and narratives – Reproductive, maternal and newborn health. (PDF)

S2 Data Detailed findings, tables and narratives – Child health. (PDF)

S1 Text Ovid MEDLINE(R) and Epub Ahead of Print, In-Process, In-Data-Review & Other Non-Indexed Citations and Daily (1946 to July 06, 2023). (PDF)

S2 Text Eligibility criteria - PICO framework. (PDF)

S1 File PRISMA checklist. (PDF)

S2 File Reflexivity checklist. (PDF)
