# Supplementary material for: Costs and cost-effectiveness of community health worker programs on reproductive, maternal, newborn and child health in low- and middle-income countries (2015–2024): A scoping review
Source: PLOS Glob Public Health. 2026 Jan 22;6(1):e0004893. doi: 10.1371/journal.pgph.0004893 (PMC12826514; doi:10.1371/journal.pgph.0004893)
Supplement: S1 Data — (PDF) [file pgph.0004893.s001.pdf]

Detailed findings, tables and narratives – Reproductive, maternal and newborn health

(i) Reproductive health

Table 1a. Details of CHW roles and scenarios

Intervention Description	Scenarios description	Role of CHW	Comparator(s)
Family Planning Counseling in Your Pocket: A Mobile Job Aid for Community Health Workers in Tanzania (1)			
A pilot intervention using a mobile job aid to guide CHWs to deliver integrated counseling on family. planning, HIV, and other sexually transmitted infections in Tanzania	One scenario (n=1)	Screened for pregnancy, counselled for contraceptives providing contraceptives or facility referral, conducting Sexually transmitted infections (STI)/HIV screening, follow-up visit for those referred to health facility.	N/A
Cost of providing injectable contraceptives through a community-based social marketing program in Tigray, Ethiopia (2)			
Community-based distribution and social marketing of injectable contraceptives in Tigray, Ethiopia.	One scenario (n=1)	CHWs were provided with microloans of 25 injections from a drug revolving fund (DRF) at the end of the training and returned to their villages to market their services through print media and community outreach.	N/A
Costs of administering injectable contraceptives through health workers and self-injection: evidence from Burkina Faso, Uganda,			

and Senegal (3)			
Community based administration of contraceptives in Uganda, Burkina Faso and Senegal.	Two scenarios: (n=2) administration of subcutaneous depot medroxyprogesterone acetate (DMPA-SC), and administration of intramuscular DMPA (DMPA-IM)	Community based distribution of DMPA-SC and DMPA-IM.	Community-based distribution of DMPA-SC (Uganda) vs self-injection of DMPA-SC (Uganda) vs facility-based delivery of DMPA-IM (Uganda) vs. facility-based delivery of DMPA-SC (Burkina Faso) vs. facility-based delivery of DMPA-IM (Burkina Faso) vs. self-injection of DMPA-SC (Senegal) vs. facility-based delivery of DMPA-IM (Senegal)
Cost-effectiveness of community-based practitioner programmes in Ethiopia, Indonesia and Kenya (4)			
CHWs in Kenya deliver MCH promotion and prevention activities and link community members to the formal health system.	One scenario (n=1)	Maternal and child health prevention and promotion activities that linked community members to the health system	Change in coverage before and after intervention

Table 1b. Summary details

Country	Type of Economic Analysis	Population served	CHWs (#)	Compensation method (2024 US$ per month)	Cost / beneficiary* (2024 US$)	Other cost outcomes **	ICER DALY (2024 US$)	Cost-effectiveness conclusion*** (threshold used)	Affordability conclusion (criteria)
Family Planning Counseling in Your Pocket: A Mobile Job Aid for Community Health Workers in Tanzania (1)									
Tanzania	Partial - cost analysis	850	25	Other (unspecified incentives per visit)	n/a	Not reported	Not reported	Not assessed	Not assessed
Cost of providing injectable contraceptives through a community-based social marketing program in Tigray, Ethiopia (2)									
Ethiopia	Partial - cost analysis	Not reported	137	No (volunteer but given microloans)	$45.51	Not reported	Not reported	Not assessed	Not assessed
Costs of administering injectable contraceptives through health workers and self-injection: evidence from Burkina Faso, Uganda, and Senegal (3)									
Burkina Faso, Uganda, Tanzania. (Only Uganda cost outcomes reported)	Partial -Cost description	Not reported	45	Other: hypothetical salary valued at nursing assistant level ($977)	Not reported	Not reported	Not reported	Cost-effective (comparison with alternative)	Not assessed
Cost-effectiveness of community-based practitioner programmes in Ethiopia, Indonesia and Kenya (4)									
Kenya	Full - CEA	5,000	3,253	No (volunteers)	Not reported	Not reported	Not reported	Cost-effective (willingness to pay)	Not assessed

* Cost per beneficiary defined as the cost per patient treated.

** Only documented cost outcomes reported in more than one study for inter-study comparison purposes, or where the cost outcome was used by the authors to determine cost effectiveness.

*** As reported by the authors. Commonly used thresholds such as GDP per capita have faced criticism for failing to consider local resource availability, such as health opportunity costs, and for being less useful in decision-making since it often results in most interventions being labelled as cost-effective.

n/a: not applicable

(ii) Maternal and newborn care

Table 2a. Details of CHW roles and scenarios

Intervention Description	Scenarios description	Role of CHW	Comparator(s)
Bolivia programme evaluation of a package to reach an underserved population: Community-based maternal and newborn care economic analysis (5)			
Community based maternal and newborn care and management of childhood illnesses in Bolivia.	Five scenarios (n=5) reporting on in different currencies and different coverage levels	Performed pregnancy visits, delivered iMCI, and occasionally attended home deliveries.	CHW package vs. 3 scale-up scenarios
Economic and cost-effectiveness analysis of the Community-Level Interventions for Pre-eclampsia (CLIP) trials in India, Pakistan and Mozambique (6)			
Community interventions for Pre-eclampsia in India, Pakistan and Mozambique.	Three scenarios (n=3); India, Pakistan and Mozambique	The intervention combined community engagement and pregnancy hypertension-focused home contacts by CHWs.	Standard care
Multi-country analysis of the cost of community health workers kits and commodities for community-based maternal and newborn care (7)			

Costs of community health worker kits and commodities for maternal and newborn care in: Bolivia, Ghana, Ethiopia, Malawi, South Africa, Tanzania and Uganda.	Seven scenarios (n=7): Bolivia, Ghana, Ethiopia, Malawi, South Africa, Tanzania and Uganda	Health promotion & clinical assessment, newborn clinical assessments and referral to a health facility if necessary.	N/A
South-Africa (Goodstart III) trial: community-based maternal and newborn care economic analysis (8)			
Home visiting intervention for maternal and newborn care in South Africa.	Three scenarios (n=3) reporting on: actual intervention, increased workload, and standardization to 100 000 mothers	Home visits. Each home visit was designated to cover specific topics with a focus on key messages.	Standard care
Overview, methods and results of multi-country community-based maternal and newborn care economic analysis (9)			
Summary papers reporting on the various Economic analyses of community-based maternal and newborn care studies.	Thirteen scenarios (n=13) reporting on interventions in: Ethiopia, Malawi, South Africa, Tanzania, Uganda, Bolivia and Ghana. Actual costs and scale up to 95% coverage	Home visiting and mobilizing activities (slightly different in the various interventions) for maternal and newborn health.	N/A
Uganda Newborn Study (UNEST) trial: Community-based maternal and newborn care economic analysis (10)			

Community based maternal and newborn intervention through home visiting in Uganda.	Four scenarios (n=4) reporting on actual intervention, gold standard coverage, increased CHW workload, and standardization to 100 000 mothers	Pregnancy and postnatal home visits (breastfeeding, hygienic cord care, and thermal protection practices).	N/A
Improving maternal and newborn care: cost-effectiveness of an innovation to rebrand traditional birth attendants in Sierra Leone (11)			
Home visiting for maternal and newborn health in Sierra Leone.	Two scenarios (n=2) reporting on rebranded traditional birth attendants, and CHWs involved in a social enterprise	Home visits including health promotion messages, maternal and newborn complication assessments, referrals to health facilities.	Standard care
The cost of the training and supervision of community health workers to improve exclusive breastfeeding amongst mothers in a cluster randomised controlled trial in South Africa (12)			
Home based support to improve breastfeeding rates in South Africa.	One scenario (n=1)	Home-based education and support to selected households and referral services.	Standard care
Malawi three district evaluation: Community-based maternal and newborn care economic analysis (13)			

Home visiting to support maternal and newborn care in Malawi.	Four scenarios (n=4) reporting on actual intervention, scale up models, and standardization to 100 000 mothers	Pregnancy and postpartum home visits, home birthing assistance, counselling on facility-based births, identification of danger signs and newborn care.	Standard care
Improving Newborn Survival in Southern Tanzania (INSIST) trial; community-based maternal and newborn care economic analysis (14)			
Home visiting to support maternal and newborn care in Tanzania.	Six scenarios (n=6) reporting on actual intervention and various modelled scale ups	CHWs visited women and their families three times in pregnancy and twice in the first few days of life.	N/A
Community-Based Interventions for Newborns in Ethiopia (COMBINE): Cost-effectiveness analysis (15)			
Home visiting to support maternal and newborn health in Ethiopia.	Sixteen scenarios (n=16) reporting on actual intervention and various modelled scale ups	Home visits, referrals, management of newborn and child illnesses, particularly bacterial infections (PSBI).	Standard care
Cost-effectiveness analysis of implementing an integrated neonatal care kit to reduce neonatal infection in rural Pakistan (16)			
CHWs distributing neonatal care kits in Pakistan.	One scenario (n=1)	LHWs provided basic reproductive, maternal, newborn and child health education and basic curative	Standard care

		care. They also distribute iNCK packages.	
Effectiveness of participatory women's groups scaled up by the public health system to improve birth outcomes in Jharkhand, eastern India: a pragmatic cluster Non-randomised controlled trial (17)			
CHWs conduct home visits and facilitate women's groups for improved birth outcomes in India.	Two scenarios (n=2) reporting in USD vs. local currency	ASHAs provided home visits, and a cycle of monthly women's group meetings.	N/A
Health and economic benefits of scaling up a home-based neonatal care package in rural India: a modelling analysis (18)			
CHWs conduct home visits for improved newborn health in India.	Two scenarios (n=2) reporting on current CHW coverage vs, increased coverage	ASHAs provided home visiting including referrals, breastfeeding support and education.	Standard care
Delivering the Thinking Healthy Programme for perinatal depression through volunteer peers: a cluster randomised controlled trial in Pakistan (19)			
CHWs deliver THPP for perinatal depression through group learning sessions.	One scenario (n=1)	CHWs delivered the THPP package, consisting of ten individual and four group sessions from the third trimester of pregnancy to 6 months after childbirth.	Standard care

Return on investments in the Health Extension Program in Ethiopia (20)			
CHWs conduct home visits for improved maternal and newborn health outcomes in Ethiopia.	One scenario (n=1)	CHWs provided primary level health promotion, prevention, and curative services to households and communities.	N/A
Cost and cost-effectiveness of newborn home visits: findings from the Newhints cluster-randomised controlled trial in rural Ghana (21)			
CHWs conduct home visits for improved newborn health in Ghana.	One scenario (n=1)	Expanded their role to include prenatal and postnatal home visits: identify pregnant women in their communities and to do two home visits.	Standard care

Table 2b. Summary details

Country	Type of Economic Analysis	Population served	CHWs (#)	Compensation method (2024 US$ per month)	Cost / beneficiary * (2024 US$)	Other cost outcomes **	ICER DALY (2024 US$)	Cost-effectiveness conclusion*** (threshold used)	Affordability conclusion (criteria)
Bolivia programme evaluation of a package to reach an underserved population: Community-based maternal and newborn care economic analysis (5)									
Bolivia	Partial - cost analysis	35,700	371	Valued at national minimum wage ($109)	$43.07 - $363.01	Cost per consultation ($11.07 - $52.91) Cost per capita ($1.6)	n/a	Not assessed	Affordable (health expenditure per capita)
Economic and cost-effectiveness analysis of the Community-Level Interventions for Pre-eclampsia (CLIP) trials in India, Pakistan and Mozambique (6)									
India	Full CEA	Not reported	148	Stipend (not documented)	$16.98	Cost per consultation ($1.88)	n/a	Mixed: overall not cost effective but high chance of being cost effective if ≥8 contacts are reached (Willingness to pay and GDP per capita)	Not assessed
Pakistan	Full CEA	Not reported	223	Salaried (not documented)	$15.42	Cost per consultation ($3.46)	n/a	Mixed: overall not cost effective but high chance of being cost effective if ≥4 contacts are reached (Willingness to pay and GDP per capita)	Not assessed

Mozambique	Full CEA	Not reported	79	Salaried (not documented)	$20.51	Cost per consultation ($4.98)	n/a	Mixed: overall not cost effective but high chance of being cost effective if ≥8 contacts are reached (Willingness to pay and GDP per capita)	Not assessed
Multi-country analysis of the cost of community health workers kits and commodities for community-based maternal and newborn care (7)									
Bolivia	Partial - cost analysis	Not documented	633	Not documented	Not reported	Cost per CHW($48)	n/a	Not assessed	Not assessed
Ghana	Partial - cost analysis	100,000	Not reported	Not documented	Not reported	Cost per CHW ($54)	n/a	Not assessed	Not assessed
Ethiopia	Partial - cost analysis	100,000	Not reported	Salaried (not documented)	Not reported	Cost per CHW ($52)	n/a	Not assessed	Not assessed
Malawi	Partial - cost analysis	100,000	Not reported	Salaried (not documented)	Not reported	Cost per CHW ($21)	n/a	Not assessed	Not assessed
South Africa	Partial - cost analysis	100,000	Not reported	Not documented	Not reported	Cost per CHW ($25)	n/a	Not assessed	Not assessed
Tanzania	Partial - cost analysis	100,000	172	No (volunteer)	Not reported	Cost per CHW ($718)	n/a	Not assessed	Not assessed
Uganda	Partial - cost analysis	100,000	Not reported	No (volunteer)	Not reported	Cost per CHW ($34)	n/a	Not assessed	Not assessed
South-Africa (Goodstart III) trial: community-based maternal and newborn care economic analysis (8)									
South Africa	Partial - cost analysis	1,894	15	Stipend ($277)	$39.44 - $101.25	Cost per consultation ($9.83 -	n/a	Not assessed	No conclusion - program

						$24.53); cost per capita ($1.00 - $2.23)			cost as share of public health expenditure would be 0-4% - 0.8%
Overview, methods and results of multi-country community-based maternal and newborn care ecomomic analysis (9)									
Ethiopia	Partial - cost analysis	Not documented	Not documented	Salary ($146)	$72.51	Cost per consultation($13.72)<br><br>Cost per capita ($0.12)	n/a	Mixed: unclear for actual CHW program, most likely cost effective CHW program scaled up to 95% coverage if the intervention averted <1 additional neonatal death (GDP per capita)	No conclusion - These financial costs amounted to $1 per capita total population, representing 5.5% of public health expenditure per capita
Malawi	Partial - cost analysis	Not documented	Not documented	Salary ($311)	$64.65	Cost per consultation($23.27)<br><br>Cost per capita ($0.59)	n/a	Mixed: unclear for actual CHW program, most likely cost effective CHW program scaled up to 95% coverage if the intervention averted <1 additional neonatal death (GDP per capita)	No conclusion - amounted to around $0.4 per capita total population Malawi and 1.3% of public health expenditure per capita

South Africa	Partial - cost analysis	Not documented	Not documented	Salary ($3216)	$101.57	Cost per consultation($24.85)<br><br>Cost per capita ($1.0)	n/a	Mixed: unclear for actual CHW program, most likely cost effective CHW program scaled up to 95% coverage if the intervention averted <1 additional neonatal death (GDP per capita)	Not assessed
Tanzania	Partial - cost analysis	Not documented	Not documented	Other (incentive per training $10.89)	$23.62	Cost per consultation($7.46)<br><br>Cost per capita ($0.32)	n/a	Mixed: unclear for actual CHW program, most likely cost effective CHW program scaled up to 95% coverage if the intervention averted <1 additional neonatal death (GDP per capita)	No conclusion - amounted to around $0.4 per capita total population in Tanzania, or 2.1% of public health expenditure per capita
Uganda	Partial - cost analysis	Not documented	Not documented	Other (incentive per meeting, $4.90)	$33.62	Cost per consultation($11.21)<br><br>Cost per capita ($0.66)	n/a	Mixed: unclear for actual CHW program, most likely cost effective CHW program scaled up to 95% coverage if the intervention averted <1 additional neonatal death (GDP per capita)	No conclusion - These financial costs amounted to $1 per capita total population in Uganda, representing 1.8% of public

									health expenditure per capita, respectively
Bolivia	Partial - cost analysis	Not documented	Not documented	No (volunteers)	$317.48	Cost per consultation($45.53)<br><br>Cost per capita ($1.06)	n/a	Mixed: unclear for actual CHW program, most likely cost effective CHW program scaled up to 95% coverage if the intervention averted <1 additional neonatal death (GDP per capita)	No conclusion - These financial costs amounted to $1 per capita total in Bolivia, representing 0.7% of public health expenditure per capita
Ghana	Partial - cost analysis	Not documented	Not documented	Stipend ($5)	$30.83	Cost per consultation($9.81)	n/a	Mixed: unclear for actual CHW program, most likely cost effective CHW program scaled up to 95% coverage if the intervention averted <1 additional neonatal death (GDP per capita)	No conclusion - amounted to around $0.4 per capita total population in Ghana or 1% of public health expenditure per capita
Uganda Newborn Study (UNEST) trial: Community-based maternal and newborn care economic analysis (10)									

Uganda	Partial - cost analysis	Not documented	50-114	Hypothetical salary ($114)	$17.20 - $44.98	Cost per consultation($4.23 - $9.92) Cost per capita ($0.70 - $1.38)	n/a	Not assessed	Not assessed
Improving maternal and newborn care: cost-effectiveness of an inNovation to rebrand traditional birth attendants in Sierra Leone (11)									
Sierra Leone	Full CEA	46 355 - 54 700	100 - 200	Other (no-interest loan in the form of a product basket valuing approximately US$27.	Not reported	Cost per capita ($13.33 - $20.25)	n/a	Mixed (one scenario: rebranded TBAs is not cost effective) (one scenario: CHWs is cost effective) (cost per capita)	Not assessed
The cost of the training and supervision of community health workers to improve exclusive breastfeeding amongst mothers in a cluster randomised controlled trial in South Africa (12)									
South Africa	Partial . cost description	736	150	Stipend (not documented)	$1 547.40	n/a	n/a	Not cost effective (comparison with alternative)	Not assessed
Malawi three district evaluation: Community-based maternal and newborn care economic analysis (13)									
Malawi	Ful CEA	34,000	533	Salaried (not documented)	$7.71 - $20.94	Cost per consultation ($1.55 - $ 7.54) Cost per capita ($0.33)	n/a	Likely cost effective (willingness to pay)	No conclusion The financial cost of universal coverage in Malawi would stand at 1.3% of

									public health expenditure if the programme is rolled out across the country.
Improving Newborn Survival in Southern Tanzania (INSIST) trial; community-based maternal and newborn care economic analysis (14)									
Tanzania	Full CEA	22,276 - 261,639	412-824	Stipend ($4)	$9.60 - $13.28	Cost per consultation ($1.79 - $3.32)<br><br>Cost per CHW ($423)<br><br>Cost per mother-child pair ($15.64)	n/a	Cost effective (comparison with alternative)	Not assessed
Community-Based Interventions for Newborns in Ethiopia (COMBINE): Cost-effectiveness analysis (15)									
Ethiopia	Full CEA	Not documented	142 - 1830	Salaried ($159)	$3.82 - $6.52	Cost per consultation ($0.45 - $1.63)<br><br>Cost per capita ($0.9 - $0.13)	$478	Cost effective (GDP per capita)	No conclusion - annualized financial costs would represent 0.3% of the country per capita public health expenditure
Cost-effectiveness analysis of implementing an integrated neonatal care kit to reduce neonatal infection in rural Pakistan (16)									

Pakistan	Full CEA	Nor documented	Not documented	Not documented	Not documented		$64	Cost effective (GDP per capita)	Not assessed
Effectiveness of participatory women's groups scaled up by the public health system to improve birth outcomes in Jharkhand, eastern India: a pragmatic cluster Non-randomised controlled trial (17)									
India	Full CEA	1,000,000	Not documented	Stipend ($14)	$0.19	Not reported	n/a	Cost-effective (GDP per capita)	Not assessed
Health and economic benefits of scaling up a home-based neonatal care package in rural India: a modelling analysis (18)									
India	Full CEA	Not documented	Not documented	Stipend (not documented)	Not reported	Cost per death averted ($510 - $514)	n/a	Cost-effective (GDP per capita)	Not assessed
Delivering the Thinking Healthy Programme for perinatal depression through volunteer peers: a cluster randomised controlled trial in Pakistan (19)									
Pakistan	Full CEA	25	Not reported	No (volunteers)	$126.29	n/a	n/a	Cost effective (willingness to pay)	Not assessed
Return on investments in the Health Extension Program in Ethiopia (20)									
Ethiopia	Full EE (social return on investment)	Not reported	37,949	Salaried (not reported)	Not reported	n/a	n/a	Cost effective (ROI)	Not assessed
Cost and cost-effectiveness of newborn home visits: findings from the Newhints cluster-randomised controlled trial in rural Ghana (21)									
Ghana	Full CEA	7,848	407	Stipend ($3)	$33.20	Cost per consultation ($10.28)<br><br>Cost per capita ($0.52)<br><br>Cost per mother and child pair ($122.54)	n/a	Cost effective (GDP per capita)	Not assessed

* Cost per beneficiary defined as the cost per patient treated.

** Only documented cost outcomes reported in more than one study for inter-study comparison purposes, or where the cost outcome was used by the authors to determine cost effectiveness.

*** As reported by the authors. Commonly used thresholds such as GDP per capita have faced criticism for failing to consider local resource availability, such as health opportunity costs, and for being less useful in decision-making since it often results in most interventions being labelled as cost-effective.

n/a: not applicable

References

1. Agarwal S, Lasway C, L'Engle K, Homan R, Layer E, Ollis S, et al. Family Planning Counseling in Your Pocket: A Mobile Job Aid for Community Health Workers in Tanzania. Glob Health Sci Pract. 2016;4(2):300-310. doi:10.9745/GHSP-D-15-00393

2. Prata N, Downing J, Bell S, Weidert K, Godefay H, Gessessew A. Cost of providing injectable contraceptives through a community-based social marketing program in Tigray, Ethiopia. Contraception. 2016 Jun 1;93(6):485–91. doi:10.1016/j.contraception.2016.01.017

3. Di Giorgio L, Mvundura M, Tumusiime J, Namagembe A, Ba A, Belemsaga-Yugbare D, et al. Costs of administering injectable contraceptives through health workers and self-injection: evidence from Burkina Faso, Uganda, and Senegal. Contraception. 2018 Nov 1;98(5):389–95. doi:10.1016/j.contraception.2018.05.018

4. McPake B, Edoka I, Witter S, Kielmann K, Taegtmeyer M, Dieleman M, et al. Cost–effectiveness of community-based practitioner programmes in Ethiopia, Indonesia and Kenya. Bull World Health Organ. 2015 Sep 1;93(9):631-639A. doi:10.2471/BLT.14.144899

5. Barger D, Pooley B, Dupuy JR, Cardenas NA, Wall S, Owen H, et al. Bolivia programme evaluation of a package to reach an underserved population: Community-based maternal and newborn care economic analysis. Health Policy Plan. 2017 Oct 1;32(suppl_1):i75–83. doi:10.1093/heapol/czv133

6. Bone JN, Khowaja AR, Vidler M, Payne BA, Bellad MB, Goudar SS, et al. Economic and cost-effectiveness analysis of the Community-Level Interventions for Pre-eclampsia (CLIP) trials in India, Pakistan and Mozambique. BMJ Glob Health. 2021 May 24;6(5). doi:10.1136/bmjgh-2020-004123

7. Barger D, Owen H, Pitt C, Kerber K, Sitrin D, Mayora C, et al. Multi-country analysis of the cost of community health workers kits and commodities for community-based maternal and newborn care. Health Policy Plan. 2017 Oct 1;32(suppl_1):i84–92. doi:10.1093/heapol/czx038

8. Daviaud E, Nkonki L, Ijumba P, Doherty T, Lawn JE, Owen H, et al. South-Africa (Goodstart III) trial: community-based maternal and newborn care economic analysis. Health Policy Plan. 2017 Oct 1;32(suppl_1):i53–63. doi:10.1093/heapol/czw112

9. Daviaud E, Owen H, Pitt C, Kerber K, Bianchi Jassir F, Barger D, et al. Overview, methods and results of multi-country community-based maternal and newborn care economic analysis. Health Policy Plan. 2017 Oct 1;32(suppl_1):i6–20. doi:10.1093/heapol/czx055

10. Elizabeth Ekirapa-Kiracho, Diana Barger, Chripus Mayora, Peter Waiswa, Joy E Lawn, James Kalungi, et al. Uganda Newborn

Study (UNEST) trial: Community-based maternal and newborn care economic analysis. Health Policy Plan. 2017 Oct 3;32(1). doi:10.1093/heapol/czw092

11. Fotso JC, Ambrose A, Hutchinson P, Ali D. Improving maternal and newborn care: cost-effectiveness of an innovation to rebrand traditional birth attendants in Sierra Leone. Int J Public Health. 2020 Dec 1;65(9):1603–12. doi:10.1007/s00038-020-01487-z

12. George G, Mudzingwa T, Horwood C. The cost of the training and supervision of community health workers to improve exclusive breastfeeding amongst mothers in a cluster randomised controlled trial in South Africa. BMC Health Serv Res. 2020 Feb 3;20(1):76. doi:10.1186/s12913-020-4913-4

13. Greco G, Daviaud E, Owen H, Ligowe R, Chimbalanga E, Guenther T, et al. Malawi three district evaluation: Community-based maternal and newborn care economic analysis. Health Policy Plan. 2017 Oct 1;32(suppl_1):i64–74. doi:10.1093/heapol/czw079

14. Manzi F, Daviaud E, Schellenberg J, Lawn JE, John T, Msemo G, et al. Improving Newborn Survival in Southern Tanzania (INSIST) trial; community-based maternal and newborn care economic analysis. Health Policy Plan. 2017 Oct;32(Suppl 1):i33–41. doi:10.1093/heapol/czw048

15. Mathewos B, Owen H, Sitrin D, Cousens S, Degefie T, Wall S, et al. Community-Based Interventions for Newborns in Ethiopia (COMBINE): Cost-effectiveness analysis. Health Policy Plan. 2017 Oct 1;32(suppl_1):i21–32. doi:10.1093/heapol/czx054

16. Muttalib F, Chung K, Pell LG, Ariff S, Soofi S, Morris SK, et al. Cost-effectiveness analysis of implementing an integrated neonatal care kit to reduce neonatal infection in rural Pakistan. 2022 Jan 1;12:e047793. doi:10.1136/bmjopen-2020-047793

17. Nair N, Tripathy PK, Gope R, Rath S, Pradhan H, Rath S, et al. Effectiveness of participatory women's groups scaled up by the public health system to improve birth outcomes in Jharkhand, eastern India: a pragmatic cluster non-randomised controlled trial. BMJ Glob Health. 2021 Nov 3;6(11). doi:10.1136/bmjgh-2021-005066

18. Nandi A, Colson AR, Verma A, Megiddo I, Ashok A, Laxminarayan R. Health and economic benefits of scaling up a home-based neonatal care package in rural India: a modelling analysis. Health Policy Plan. 2016 Jun 1;31(5):634–44. doi:10.1093/heapol/czv113

19. Sikander S, Ahmad I, Atif N, Zaidi A, Vanobberghen F, Weiss HA, et al. Delivering the Thinking Healthy Programme for perinatal depression through volunteer peers: a cluster randomised controlled trial in Pakistan. Lancet Psychiatry. 2019 Feb 1;6(2):128–39. doi:10.1016/S2215-0366(18)30467-X

20. Bowser D, Kleinau E, Berchtold G, Kapaon D, Kasa L. Return on investments in the Health Extension Program in Ethiopia. PLOS ONE. 2023 Nov 27;18(11):e0291958. doi:10.1371/journal.pone.0291958

21. Pitt C, Tawiah T, Soremekun S, Asbroek AHA ten, Manu A, Tawiah-Agyemang C, et al. Cost and cost-effectiveness of newborn home visits: findings from the Newhints cluster-randomised controlled trial in rural Ghana. Lancet Glob Health. 2016 Jan 1;4(1):e45–56. doi:10.1016/S2214-109X(15)00207-7
