# Supplementary material for: Costs and cost-effectiveness of community health worker programs on reproductive, maternal, newborn and child health in low- and middle-income countries (2015–2024): A scoping review
Source: PLOS Glob Public Health. 2026 Jan 22;6(1):e0004893. doi: 10.1371/journal.pgph.0004893 (PMC12826514; doi:10.1371/journal.pgph.0004893)
Supplement: S2 Data — (PDF) [file pgph.0004893.s002.pdf]

Detailed findings, tables and narratives – Child health

(i) Child mortality and survival

Table 1a. Details of CHW roles and scenarios

Intervention Description	Scenarios descriptions	Role of CHW	Comparator
**Study title:** Trial-based economic evaluation of the system-integrated activation of community health volunteers in rural Ghana (22)			
Community-based primary healthcare intervention for surveillance of disease among children under 5 years of age in Ghana.	One scenario (n=1)	Completed regular home visits for health promotion and diarrhoea and high fever surveillance.	Government-paid community health officers vs. community health volunteers
**Study title:** Modelling the cost of community interventions to reduce child mortality in South Africa using the Lives Saved Tool (LiST) (23)			
Community-based interventions to reduce child mortality in South Africa.	One scenario (n=1)	Delivered intervention and education in homes.	All delivery channels
**Study title:** Can Community Health Workers Report Accurately on Births and Deaths? Results of Field Assessments in Ethiopia, Malawi and Mali (24)			
Reporting counts of births and	Three scenarios (n=3):	Reported pregnancies, births	Not assessed

deaths and under-five, infant, and neonatal mortality rates in Ethiopia, Malawi and Mali.	Ethiopia, Malawi, Mali	and deaths within defined geographic areas over a period of at least fifteen months.	
**Study title:** Comparison of door-to-door and fixed-point delivery of azithromycin distribution for child survival in Niger: A cluster-randomized trial (25)			
Biannual distribution of azithromycin to combat infections in children under 5 in Niger.	Four scenarios (n=4): two rounds using fixed-point delivery, two rounds distributing door-to-door	Delivered a single dose of oral azithromycin to children 1–59 months biannually through a door-to-door delivery approach.	Door-to-door vs. fixed-point delivery

Table 1b. Summary details

Country	Type of Economic Analysis	Population served	CHWs (#)	Compensation method (2024 US$ per month)	Cost / beneficiary* (2024 US$)	Other cost outcomes **	ICER DALY (2024 US$)	Cost-effectiveness conclusion*** (threshold used)	Affordability conclusion (criteria)
Trial-based economic evaluation of the system-integrated activation of community health volunteers in rural Ghana (22)									
Ghana	Full CEA	24,765	Not reported	Salaried (not documented)	Not reported	n/a	$2,220.25	Mixed - activating CHVs in Ghana's health system to induce disease reduction in children through home visits meets the WHO-CHOICE thresholds. (GDP per Capita)	No conclusion - GDP/capita thresholds cannot be viewed as simple indicators of economic feasibility due to the lack of a rigorous analysis of Ghana's national budget constraints
Modelling the cost of community interventions to reduce child mortality in South Africa using the Lives Saved Tool (LiST) (23)									
South Africa	Partial - cost analysis	Not reported	Not reported	Salaried (not documented)	Not reported	Cost per capita ($0.40)	Not reported	Not assessed	Yes - considered to be well within the scope and affordability of the SA health budget.
Can Community Health Workers Report Accurately on Births and Deaths? Results of Field Assessments in Ethiopia, Malawi and Mali (24)									

Ethiopia	Partial - cost analysis	509,395	183	Stipend ($72)	$147.15	Cost per capita ($1.07)	Not reported	Not assessed	Not assessed
Malawi	Partial - cost analysis	293,741	160	Stipend ($268)	Not reported	Cost per capita ($1.16) Cost/service ($56.28)	Not reported	Not assessed	Not assessed
Mali	Partial - cost analysis	32,128	78	Not documented	Not reported	Cost per capita ($6.14) Cost/service ($144.22)	Not reported	Not assessed	Not assessed
Comparison of door-to-door and fixed-point delivery of azithromycin distribution for child survival in Niger: A cluster-randomized trial (25)									
Niger	Partial - cost analysis	55,212	45-47	Stipend (not documented)	$3.07	n/a	Not reported	Not assessed	Not assessed

* Cost per beneficiary defined as the cost per patient treated.

** Only documented cost outcomes reported in more than one study for inter-study comparison purposes, or where the cost outcome was used by the authors to determine cost effectiveness.

*** As reported by the authors. Commonly used thresholds such as GDP per capita have faced criticism for failing to consider local resource availability, such as health opportunity costs, and for being less useful in decision-making since it often results in most interventions being labelled as cost-effective.

n/a: not applicable

(ii) Child development

Table 2a. Details of CHW roles and scenarios

Intervention Description	Scenarios descriptions	Role of CHW	Comparator
Cost-effectiveness and economic returns of group-based parenting interventions to promote early childhood development: Results from a randomized controlled trial in rural Kenya (26)			
Early child development parenting intervention for families with children under age three in Kenya.	Two scenarios (n=2): group only sessions, mixed-method delivery combining group sessions with home visits	Facilitated group parent educational sessions and conduct home visits.	Group only approach vs. mixed-method approach vs. standard care
Mentor Mothers Program Improved Child Health Outcomes At A Relatively Low Cost In South Africa (27)			
Female CHW network providing home visits, health screenings, immunizations, and support for mothers and children aged 6 and under in South Africa.	Two scenarios (n=2): intervention, scale-up	Conducted pre- and postnatal home visits once a month to all pregnant community members. Visited all households in their catchment area with children age 6 and younger. Weighed children, encouraged clinical care for people living with HIV and determined whether childhood immunizations have been received.	Intervention vs. standard care vs. upscale in efficiency
Measuring the cost-effectiveness of a home-visiting intervention to promote early child development among rural families linked to the Rwandan social protection system (28)			
Home-visiting program designed to improve early child development and reduce family violence in in Rwanda.	Three scenarios (n=3): various forms of cash for work schemes, including a basic scheme of cash for work, and expanded schemes including financial literacy and asset transfers.	Provided information on health and education during weekly visits to participants' homes. Facilitated cash-for-work program.	Various forms of cash for work schemes, including a basic scheme of cash for work, and expanded schemes including financial literacy and asset transfer.

Table 2b. Summary details

Country	Type of Economic Analysis	Population served	CHWs (#)	Compensation method (2024 US$ per month)	Cost / beneficiary* (2024 US$)	Other cost outcomes **	ICER DALY (2024 US$)	Cost-effectiveness conclusion*** (threshold used)	Affordability conclusion (criteria)
Cost-effectiveness and economic returns of group-based parenting interventions to promote early childhood development: Results from a randomized controlled trial in rural Kenya (26)									
Kenya	Full CEA	1,070	20	Stipend ($20)	$139.72 - $144.71	n/a	Not reported	Cost-effective (benefit-cost ratio and return on investment)	Not assessed
Mentor Mothers Program Improved Child Health Outcomes At A Relatively Low Cost In South Africa (27)									
South Africa	Full CEA	1,238	9-12	Salaried ($312)	$110.13 - $145.27	n/a	Not reported	Not assessed	Yes - employment of community health workers was cost saving compared to that of nurses. (comparison with alternative)
Measuring the cost-effectiveness of a home-visiting intervention to promote early child development among rural families linked to the Rwandan social protection system (28)									
Rwanda	Full CEA	1,498	Not reported	Not documented	$210.54 - $482.45	Cost/consultation ($17.99 - $40.20)	Not reported	Mixed scenario a and b not cost effective, scenario c is cost effective (comparison with alternative)	Not assessed

* Cost per beneficiary defined as the cost per patient treated.

** Only documented cost outcomes reported in more than one study for inter-study comparison purposes, or where the cost outcome was used by the authors to determine cost effectiveness.

*** As reported by the authors. Commonly used thresholds such as GDP per capita have faced criticism for failing to consider local resource availability, such as health opportunity costs, and for being less useful in decision-making since it often results in most interventions being labelled as cost-effective.

n/a: not applicable

(iii) Child nutrition

Table 3a. Details of CHW roles and scenarios

Intervention Description	Scenarios description	Role of CHW	Comparator
Mothers' screening for malnutrition by mid-upper arm circumference is Non-inferior to community health workers: results from a large-scale pragmatic trial in rural Niger (29)			
CHWs screening and referring for malnutrition in Niger.	One scenario (n=1)	Raised awareness about the signs of malnutrition during screenings, and gave referral slips to the mothers of children indicating why the child was referred.	CHWs vs. mothers
Costs, cost-effectiveness, and financial sustainability of CMAM in Northern Nigeria (30)			
Community management of acute malnutrition in Nigeria.	One scenario (n=1)	Performed community management of acute malnutrition (CMAM).	n/a
Social value of a nutritional counselling and support program for breastfeeding in urban poor settings, Nairobi (31)			
Nutritional counselling and support for breastfeeding in Kenya.	One scenario (n=1)	Home-based counselling of pregnant and breastfeeding women and mothers of young children on optimal practices.	Standard care
Cost effectiveness of a community based prevention and treatment of acute malnutrition programme in Mumbai slums, India (32)			
Community management of acute malnutrition in India.	One scenario (n=1)	Prevented and treated acute malnutrition in infants under 3 years old, focused on increasing optimal breastfeeding practices and improving complementary feeding practices.	Standard care
Integration of acute malnutrition treatment into integrated community case management in three districts in Southern Mali: an economic evaluation (33)			

Integration of acute malnutrition treatment into ICCM in Mali.	Six scenarios (n=6) reporting outcomes for cost per child treated and cost per child recovered in three districts. Scenarios also reporting on various CHW supervision strategies	Integrated treatment of acute malnutrition in their standard ICCM work.	Various CHW supervision strategies
The costs of Suaahara II, a complex scaled-up multi sectoral nutrition programme in Nepal (34)			
Expanded multisectoral nutrition strategies in Nepal.	Four scenarios (n=4) reporting on 1) overall, 2) terai agro ecological zone, 3) hill agro ecological zone and 4) mountain agro ecological zone	Targeted disadvantaged households with an enhanced set of interventions including the addition of homestead food production. Included home visits and community events designed to improve access to and demand for nutritious foods.	Different districts/agro ecological zones
Treatment of moderate acute malnutrition through community health volunteers is a cost-effective intervention: Evidence from a resource-limited setting (35)			
CHWs treating moderate acute malnutrition in Kenya.	Two scenarios (n=2), one reporting on costs per child treated and one cost per child recovered	Sought and screened children with acute malnutrition in the community through house-to-house visits.	CHVs plus health facility treatment vs. health facility treatment alone
Evaluation of the cost-effectiveness of the treatment of uncomplicated severe acute malnutrition by lady health workers as compared to an outpatient therapeutic feeding programme in Sindh Province, Pakistan (36)			
Treatment of uncomplicated severe acute malnutrition by CHWs in Pakistan.	One scenario (n=1)	Screened for severe acute malnutrition (SAM), treated cases without medical complications and referred any complicated cases to the health centre.	CHWs vs. outpatient feeding program
Cost-effectiveness of the treatment of uncomplicated severe acute malnutrition by community health workers compared to treatment provided at an outpatient facility in rural Mali (37)			
Treatment of uncomplicated severe acute malnutrition by CHWs in Mali.	Two scenarios (n=2): 1) costs per child treated, 2) costs per child recovered	Screened for SAM, referred complicated cases, treated uncomplicated cases in communities and provided nutrition information sessions to communities.	Standard care
Effectiveness of screening and treatment of children with severe acute malnutrition by community health workers in Simiyu region, Tanzania: a quasi-experimental pilot study (38)			

CHWs screening and treating SAM in Tanzania.	Two scenarios (n=2): 1) costs per child treated, 2) costs per child recovered	Screened children for SAM by measuring middle upper arm circumference (MUAC) and treated or referred depending on the need.	CHW treatment vs referral to treatment at facility
Cost-effectiveness of severe acute malnutrition treatment delivered by community health workers in the district of Mayahi, Niger (39)			
CHWs screening and treating SAM in Niger.	Two scenarios (n=2): 1) costs per child treated, 2) costs per child recovered	Screened for and treated SAM.	Standard care
Cost-effectiveness of a market-based home fortification of food with micronutrient powder programme in Bangladesh (40)			
Micronutrient powder distributed by CHWs in Bangladesh.	One scenario (n=1)	Counselled on infant and young child feeding and home fortification and sold micronutrients.	Standard care
Comparing costs and cost-efficiency of platforms for micronutrient powder (MNP) delivery to children in rural Uganda (41)			
Various platforms for micronutrient powder distribution in Uganda.	Six scenarios (n=6) reporting on various management strategies for the program, including NGO management, MoH takeover, and volunteer or paid CHWs	Reminded caregivers of children 6–23 months old to pick up MNP packets.	Delivery through CHWs vs. health facilities

Table 3b. Summary details

Country	Type of Econ. Analysis	Pop. served	CHWs (#)	Compensation method (2024 US$ per month)	Cost/ beneficiary* (2024 US$)	Cost/child recovered (2024 US$)	Other cost outcome** (2024 US$)	Cost-effectiveness conclusion*** (threshold used)	Affordability conclusion (criteria)
Mothers' screening for malnutrition by mid-upper arm circumference is non-inferior to community health workers: results from a large-scale pragmatic trial in rural Niger (29)									
Niger	Partial - Cost descriptive	8,867	36	Stipend ($46)	$3.17	Not assessed	Not reported	Not cost effective (compared with alternative)	Not assessed
Costs, cost-effectiveness, and financial sustainability of CMAM in Northern Nigeria (30)									
Nigeria	Full CEA	Not reported	719	Stipend (not documented)	Not reported	Cost/child recovered ($371)	Cost/DALY averted $51;<br><br>cost/death averted ($1,891)	Highly cost effective (GDP per capita)	Affordable - (health budget, GDP per capita)
Social value of a nutritional counselling and support program for breastfeeding in urban poor settings, Nairobi (31)									
Kenya	Social return on investment	1,100	Not reported	Salaried (not documented)	Not reported	Not reported	Not reported	Not assessed	Not assessed
Cost effectiveness of a community-based prevention and treatment of acute malnutrition programme in Mumbai slums, India (32)									
India	Full EE - CEA and CUA	30	30	No (volunteers)	$32.32	Not reported	Cost/DALY averted ($28); cost per death averted ($15,117)	Cost-effective (GDP per capita)	Not assessed
Integration of acute malnutrition treatment into integrated community case management in three districts in Southern Mali: an economic evaluation (33)									

Mali	Partial - Cost Description	233,647 - 513,172	30 - 68	Salaried (not documented)	$213.09 - $340.37	Not reported	Not reported	Not assessed	Not assessed
The costs of Suaahara II, a complex scaled-up multi sectoral nutrition programme in Nepal (34)									
Nepal	Partial - description	9,245 - 15,005	Not documented	Stipend ($150)	$78.92 - $95.91	Not reported	Cost/ mother-child pair ($78.9 - $92.2)	Not assessed	No conclusion
Treatment of moderate acute malnutrition through community health volunteers is a cost-effective intervention: Evidence from a resource-limited setting (35)									
Kenya	Full CEA	272	61	No (volunteers)	$215.45 - $308.08	Cost per child recovered ($308)	Cost per DALY averted ($400); cost per child treated ($215); cost per death averted ($8,802)	Cost effective (comparison with alternative)	No conclusion
Evaluation of the cost-effectiveness of the treatment of uncomplicated severe acute malnutrition by lady health workers as compared to an outpatient therapeutic feeding programme in Sindh Province, Pakistan (36)									
Pakistan	Full CEA	Not reported	72	Salaried (not documented)	$380.18	Cost per child cured ($499)	Cost per child treated ($380)	Not cost effective (comparison with alternative)	Not assessed
Cost-effectiveness of the treatment of uncomplicated severe acute malnutrition by community health workers compared to treatment provided at an outpatient facility in rural Mali (37)									
Mali	Full CEA	Not reported	34	Salaried (not documented)	$318.78 - $338.88	Cost per child recovered ($338)	Cost per child treated ($318);	Cost effective (comparison with alternative)	Not assessed
Effectiveness of screening and treatment of children with severe acute malnutrition by community health workers in Simiyu region, Tanzania: a quasi-experimental pilot study (38)									
Tanzania	Full CEA	154	13	Stipend (not reported)	$176.48 - $194.87	Cost per child cured ($194)	Cost per child treated ($176);	Cost effective (comparison with alternative)	Not assessed
Cost-effectiveness of severe acute malnutrition treatment delivered by community health workers in the district of Mayahi, Niger (39)									

Niger	Full CEA	1,977	Not reported	Salaried ($103)	$98.26 - $127.22	Not reported	Cost per service ($6.06 - $10.10)	Cost effective (comparison with alternative)	Not assessed
Cost-effectiveness of a market-based home fortification of food with micronutrient powder programme in Bangladesh (40)									
Bangladesh	Full CEA	5,358,604	Not reported	No (volunteers)	Not reported	Not reported	Cost per DALY averted ($195)	Cost-effective (GDP per capita)	Not assessed
Comparing costs and cost-efficiency of platforms for micronutrient powder (MNP) delivery to children in rural Uganda (41)									
Uganda	Partial - cost description	1,072	Not reported	Stipend ($4/day)	$49.24 - $72.71	Not reported	Cost per child reached/treated ($49.23 - $72.12)	Cost effective (comparison with alternative)	Not assessed

* Cost per beneficiary defined as the cost per patient treated.

** Only documented cost outcomes reported in more than one study for inter-study comparison purposes, or where the cost outcome was used by the authors to determine cost effectiveness.

*** As reported by the authors. Commonly used thresholds such as GDP per capita have faced criticism for failing to consider local resource availability, such as health opportunity costs, and for being less useful in decision-making since it often results in most interventions being labelled as cost-effective.

n/a: not applicable

(iv) Childhood infectious disease prevention and management

Table 4a. Details of CHW roles and scenarios

Intervention Description	Scenarios descriptions	Role of CHW	Comparator
Are community health workers cost-effective for childhood vaccination in India? (42)			
Female CHWs provide measles vaccinations and support pregnant individuals in India	One scenario (=1)	Accompanied pregnant women for delivery, take infants and children for immunization, refer children with life-threatening conditions, deliver basic antenatal and postnatal care/advice.	Intervention vs. standard care at health facility
Costs of implementing integrated community case management (iCCM) in six African countries: implications for sustainability (43)			
Integrated community case management of childhood malaria, diarrhea and pneumonia in Ethiopia, Ghana, Mali, Malawi, Mozambique, and Niger.	Six scenarios (n=6): Ethiopia, Ghana, Mali, Malawi, Mozambique, and Niger.	Provided iCCM treatment, diagnosis and management for malaria, pneumonia and diarrhea.	n/a
Cost-effectiveness analysis of the national implementation of integrated community case management and community-based health planning and services in Ghana for the treatment of malaria, diarrhoea and pneumonia (44)			
Integrated community case management of childhood malaria, diarrhea and pneumonia in Ghana.	Six scenarios (n=6): malaria, diarrhea and pneumonia, each reported in two districts	Diagnosed, educated, monitored and referred cases of malaria, diarrhea and pneumonia	Intervention vs. standard care at health facility
Valuing the work of unpaid community health workers and exploring the incentives to volunteering in rural Africa (45)			

Integrated community case management of malaria, pneumonia and diarrhea for children under 5 years of age in Uganda	One scenario (n=1)	Provided diagnosis, treatment and referrals for malaria pneumonia and diarrhea.	Opportunity cost of CHW time vs. lowest wage rate in the health system in Uganda
Estimating the cost of referral and willingness to pay for referral to higher-level health facilities: a case series study from an integrated community case management programme in Uganda (46)			
Integrated community case management of malaria, pneumonia, and diarrhea for children in Uganda.	Four scenarios (n=4): intervention and referral services across three distinct levels of health facilities	Diagnosed and treated children with uncomplicated disease, whilst referring those with danger signs.	Intervention vs. referral to three levels of health facilities
Cost-effectiveness analysis of integrated community case management delivery models utilizing drug sellers and community health workers for treatment of under-five febrile cases of malaria, pneumonia, diarrhoea in rural Uganda (47)			
Integrated community case management of malaria, pneumonia, and diarrhea for children under five in Uganda.	Three scenarios (n=3): malaria, pneumonia, diarrhea	Diagnosis and treatment of malaria, pneumonia, and diarrhoea in children under 5.	iCCM-trained drug sellers vs. iCCM-trained CHWs vs. Non-iCCM-trained CHWs
Patient-level cost of home- and facility-based child pneumonia treatment in Suba Sub County, Kenya (48)			
Community-based pneumonia treatment for children in Kenya.	One scenario (n=1)	Pneumonia community case management, including treatment of mild pneumonia.	Intervention vs. standard care at a health facility
Cost-Effectiveness Analysis of Community Case Management of Childhood Diarrhea in Burundi. Provider perspective (49)			
Community-based diarrhea treatment for children in Burundi.	Two scenarios (n=2): societal perspective, provider perspective	Provided primary treatment for pneumonia in children under the age of 5 years.	Intervention vs. standard care at a health facility
Reducing regional health inequality: a sub-national distributional cost-effectiveness analysis of community-based treatment of childhood pneumonia in Ethiopia (50)			

Community-based treatment of childhood pneumonia in Ethiopia.	Four scenarios (n=4): various scale-ups	Educational preventative work alongside treatment of pneumonia	Intervention vs. standard care at a health facility
Impact and efficiency of the integration of diagnosis and treatment of pneumonia in the community management of malaria in Madagascar (51)			
Community-based treatment of childhood pneumonia and malaria in Madagascar.	One scenario (n=1)	Detected and treated cases of pneumonia.	Intervention vs. basic community health activities
The cost-effectiveness of community health workers delivering free diarrhoea treatment: evidence from Uganda (52)			
Community-based diarrhea treatment distribution with free and sales model in Uganda.	Six scenarios (n=6): free door-to-door ORS distribution, facility-based sales distribution door-to-door sales distribution of ORS. Each reported in an implementer and societal perspective.	Home visits to households that contained a child under 5 to distribute ORS and zinc to store in their homes.	Free distribution facility based sales distribution vs. door-to-door sales distribution vs. basic community health activities
Cost-effectiveness of village health worker-led integrated community case management (iCCM) versus health facility based management for childhood illnesses in rural southwestern Uganda (53)			
iCCM of childhood illnesses, including malaria, diarrhea, and pneumonia, in Uganda.	Three scenarios: (n=3) malaria, diarrhea, pneumonia.	Identified, treated, and/or referred children under five who have diarrhoea, pneumonia, or malaria.	Intervention vs. standard care at health facility

Table 2h. Summary Details – Childhood Infectious Disease Prevention and Management

Country	Type of Economic Analysis	Population served	CHWs (#)	Compensation method (2024 US$ per month)	Cost / beneficiary* (2024 US$)	Cost per capita per year (2024 US$)	Other cost outcomes **	Cost-effectiveness conclusion*** (threshold used)	Affordability conclusion (criteria)
Are community health workers cost-effective for childhood vaccination in India? (42)									
India	Full CEA	Not reported	Not reported	No (volunteers)	Not reported	Not reported	Cost/DALY averted ($206)	Cost-effective (GDP per capita)	Not assessed
Costs of implementing integrated community case management (iCCM) in six African countries: implications for sustainability (43)									
Ethiopia	Partial - cost analysis	Not reported	27,116	Salaried ($78)	Not reported	Cost/capita per year ($0.12)	Total cost/CHW ($310);	Not assessed	Inconclusive (percentage of public health expenditure per capita) - <0.6%
Ghana	Partial - cost analysis	Not reported	16,812	No	Not reported	Cost/capita per year ($0.28)	Total cost/CHW ($142); cost/consultation ($12.61)	Not assessed	Inconclusive (percentage of public health expenditure per capita) - <0.6%
Mali	Partial - cost analysis	Not reported	1,847	Stipend ($91)	Not reported	Cost/capita per year ($0.65)	Total cost/CHW ($1,209);	Not assessed	Inconclusive (percentage of public health expenditure per capita) - 2.7%
Malawi	Partial - cost analysis	Not reported	1,018	Salaried ($284)	Not reported	Cost/capita per year ($0.59)	Total cost/CHW	Not assessed	Inconclusive (percentage of of public

							($2,079); Cost/consultation ($0.26)		health expenditure per capita) - 1.8%
Mozambique	Partial - cost analysis	Not reported	905	Stipend ($48)	Not reported	Cost/capita per year ($0.13)	Total cost/CHW ($549)	Not assessed	Inconclusive (percentage of of public health expenditure per capita)- <0.6%
Niger	Partial - cost analysis	Not reported	2,560	Salaried ($49)	Not reported	Cost/capita per year ($0.89)	Total cost/CHW ($2,464);	Not assessed	Inconclusive (percentage of public health expenditure per capita) - 7.4%
Cost-effectiveness analysis of the national implementation of integrated community case management and community-based health planning and services in Ghana for the treatment of malaria, diarrhoea and pneumonia (44)									
Ghana	Full CEA	1,900,000 -2,500,00	920-5,000	Other (non-monetary incentive package including bicycles and clothing)	$0.50 - $10.33	Not reported	n/a	Cost-effective (comparison with alternative)	Not assessed
Valuing the work of unpaid community health workers and exploring the incentives to volunteering in rural Africa (45)									
Uganda	Discrete Choice Experiment	Not reported	45	No	Not reported	Not reported	n/a	Not assessed	Not assessed
Estimating the cost of referral and willingness to pay for referral to higher-level health facilities: a case series study from an integrated community									

case management programme in Uganda (46)									
Uganda	Partial - cost description	2,200,000	Not reported	No	$5.84 - $8.99	Cost/capita per year ($1.55 - $4.05)	n/a	Not assessed	Not assessed
Cost-effectiveness analysis of integrated community case management delivery models utilizing drug sellers and community health workers for treatment of under-five febrile cases of malaria, pneumonia, diarrhoea in rural Uganda (47)									
Uganda	Full CEA	Not reported	Not reported	Other (non-monetary incentive package including rainboots and solar lights)	Not reported	Not reported	Cost/CHW per year ($231 - $1,286)	Cost-effective (willingness to pay)	Mixed
Patient-level cost of home- and facility-based child pneumonia treatment in Suba Sub County, Kenya (48)									
Kenya	Partial - cost analysis	17,900	Not reported	No (volunteers)	$123.48	Not reported	n/a	Cost effective (comparison with alternative)	Not assessed
Cost-Effectiveness Analysis of Community Case Management of Childhood Diarrhea in Burundi. Provider perspective (49)									
Burundi	Full CEA	Not reported	Not reported	No (volunteers)	$9.14	Not reported	n/a	Cost-effective (GDP per capita)	Not assessed
Reducing regional health inequality: a sub-national distributional cost-effectiveness analysis of community-based treatment of childhood pneumonia in Ethiopia (50)									
Ethiopia	Full CEA	Not reported	Not reported	Salaried (not documented)	Not reported	Not reported	n/a	Cost-effective (GDP per capita) - Regional variation	Not assessed
Impact and efficiency of the integration of diagnosis and treatment of pneumonia in the community management of malaria in Madagascar (51)									

Madagascar	Full CEA	54,952	201	No (volunteers)	$7.48	Not reported	Total cost/CHW ($1,301)	Inconclusive (per capita health expenditure) - insufficient data on program effectiveness	Not affordable
The cost-effectiveness of community health workers delivering free diarrhoea treatment: evidence from Uganda (52)									
Uganda	Full CEA	2,363	118	Not documented	$0.02 - $0.58	Not reported	Cost/DALY averted ($9-$13)	Mixed (two scenarios are cost effective two unlikely and two not cost effective). (comparison with alternative, GDP per capita)	Not assessed
Cost-effectiveness of village health worker-led integrated community case management (iCCM) versus health facility based management for childhood illnesses in rural southwestern Uganda (53)									
Uganda	Full CEA	45,000	Not reported	Other (non-monetary incentive package)	$2.55 - $14.19	Not reported	n/a	Cost-effective (willingness to pay)	Not assessed

* Cost per beneficiary defined as the cost per patient treated.

** Only documented cost outcomes reported in more than one study for inter-study comparison purposes, or where the cost outcome was used by the authors to determine cost effectiveness.

*** As reported by the authors. Commonly used thresholds such as GDP per capita have faced criticism for failing to consider local resource availability, such as health opportunity costs, and for being less useful in decision-making since it often results in most interventions being labelled as cost-effective.

n/a: not applicable

References

22. Cho Y, Awoonor-Williams ,Koku, Jun ,Damin, Oh ,Chunghyeon, and Cha S. Trial-based economic evaluation of the system-integrated activation of community health volunteers in rural Ghana. Glob Health Action. 2023 Dec 31;16(1):2203541. doi: 10.1080/16549716.2023.2203541

23. Lungiswa LN, Lumbwe LC, Aviva AT, Karen KH. Modelling the cost of community interventions to reduce child mortality in South Africa using the Lives Saved Tool (LiST). 2017. doi: 10.1136/bmjopen-2016-011425

24. Silva R, Amouzou A, Munos M, Marsh A, Hazel E, Victora C, et al. Can Community Health Workers Report Accurately on Births and Deaths? Results of Field Assessments in Ethiopia, Malawi and Mali. PLOS ONE. 2016 Jan 5;11(1):e0144662. doi: 10.1371/journal.pone.0144662

25. Arzika AM, Maliki R, Amza A, Karamba A, Gallo N, Aichatou B, et al. Comparison of door-to-door and fixed-point delivery of azithromycin distribution for child survival in Niger: A cluster-randomized trial. PLOS Glob Public Health. 2023 Nov 15;3(11):e0002559. doi: 10.1371/journal.pgph.0002559

26. Garcia IL, Saya UY, Luoto JE. Cost-effectiveness and economic returns of group-based parenting interventions to promote early childhood development: Results from a randomized controlled trial in rural Kenya. PLOS Med. 2021 Sep 28;18(9):e1003746. doi: 10.1371/journal.pmed.1003746

27. Wynn A, Rotheram-Borus MJ, Leibowitz AA, Weichle T, Roux I le, Tomlinson M. Mentor Mothers Program Improved Child Health Outcomes At A Relatively Low Cost In South Africa. Health Aff (Millwood) [Internet]. 2017 Nov [cited 2025 Mar 30];36(11):1947–55. doi: 10.1377/hlthaff.2017.0553

28. Desmond C, Watt KG, Jensen SKG, Simmons E, Murray SM, Farrar J, et al. Measuring the cost-effectiveness of a home-visiting intervention to promote early child development among rural families linked to the Rwandan social protection system. PLOS Glob Public Health. 2023 Oct 24;3(10):e0002473. doi: 10.1371/journal.pgph.0002473

29. Alé FGB, Phelan KPQ, Issa H, Defourny I, Le Duc G, Harczi G, et al. Mothers screening for malnutrition by mid-upper arm circumference is non-inferior to community health workers: results from a large-scale pragmatic trial in rural Niger. Arch Public Health. 2016 Sep 6;74(1):38. doi: 10.1186/s13690-016-0149-5

30. Frankel S, Roland M, Makinen M. Costs, cost-effectiveness, and financial sustainability of CMAM in Northern Nigeria [Internet]. Field Exchange. 2015 [cited 2025 Mar 30]. Available from: https://www.ennonline.net/fex/50/en/costs-cost-effectiveness-and-financial-sustainability-cmam-northern-nigeria

31. Goudet S, Griffiths PL, Wainaina CW, Macharia TN, Wekesah FM, Wanjohi M, et al. Social value of a nutritional counselling and support program for breastfeeding in urban poor settings, Nairobi. BMC Public Health. 2018 Apr 2;18(1):424. doi: 10.1186/s12889-018-5334-8

32. Goudet S, Jayaraman A, Chanani S, Osrin D, Devleesschauwer B, Bogin B, et al. Cost effectiveness of a community based prevention and treatment of acute malnutrition programme in Mumbai slums, India. PloS One. 2018;13(11):e0205688. doi: 10.1371/journal.pone.0205688

33. Cichon B, López-Ejeda N, Mampindu MB, Bagayoko A, Samake M, Cuellar PC. Integration of acute malnutrition treatment into integrated community case management in three districts in Southern Mali: an economic evaluation. Glob Health Sci Pract. 2024 Jun 27;12(3). doi: 10.9745/GHSP-D-23-00431

34. Choo EM, Kemp CG, Sagun KC, Paudel U, Wun J, Cunningham K, et al. The costs of Suaahara II, a complex scaled-up multisectoral nutrition programme in Nepal. Matern Child Nutr. 2024 May 5. doi: 10.1111/mcn.13658

35. Ilboudo PG, Donfouet HPP, Wilunda C, Cichon B, Tewoldeberhan D, Njiru J, et al. Treatment of moderate acute malnutrition through community health volunteers is a cost-effective intervention: Evidence from a resource-limited setting. Matern Child Nutr. 2024;20(4):e13695. doi: 10.1111/mcn.13695

36. Rogers E, Guerrero S, Kumar D, Soofi S, Fazal S, Martínez K, et al. Evaluation of the cost-effectiveness of the treatment of uncomplicated severe acute malnutrition by lady health workers as compared to an outpatient therapeutic feeding programme in Sindh Province, Pakistan. BMC Public Health. 2019 Jan 17;19(1):84. doi: 10.1186/s12889-018-6382-9

37. Rogers E, Martínez K, Morán JLA, Alé FGB, Charle P, Guerrero S, et al. Cost-effectiveness of the treatment of uncomplicated severe acute malnutrition by community health workers compared to treatment provided at an outpatient facility in rural Mali. Hum Resour Health. 2018 Feb 20;16(1):12. doi: 10.1186/s12960-018-0273-0

38. Wilunda C, Mumba FG, Putoto G, Maya G, Musa E, Lorusso V, et al. Effectiveness of screening and treatment of children with severe acute malnutrition by community health workers in Simiyu region, Tanzania: a quasi-experimental pilot study. Sci Rep. 2021 Jan 27;11(1):2342. doi: 10.1038/s41598-021-81811-6

39. Molanes-López EM, Ferrer JM, Dougnon AO, Gado AA, Sanoussi A, Ousmane N, et al. Cost-effectiveness of severe acute malnutrition treatment delivered by community health workers in the district of Mayahi, Niger. Hum Resour Health. 2024 Mar 29;22(1):22. doi: 10.1186/s12960-024-00904-1

40. Ahmed S, Sarma H, Hasan Z, Rahman M, Ahmed MW, Islam MA, et al. Cost-effectiveness of a market-based home fortification of food with micronutrient powder programme in Bangladesh. Public Health Nutr. 2021 Apr;24(S1):s59–70. doi:

10.1017/S1368980020003602

41. Schott W, Richardson B, Baker E, D'Agostino A, Namaste S, Vosti SA. Comparing costs and cost-efficiency of platforms for micronutrient powder (MNP) delivery to children in rural Uganda. Ann N Y Acad Sci. 2021;1502(1):28–39. doi: 10.1111/nyas.14621

42. Bettampadi D, Boulton ML, Power LE, Hutton DW. Are community health workers cost-effective for childhood vaccination in India? Vaccine. 2019 May 16;37(22):2942–51. doi: 10.1016/j.vaccine.2019.04.038

43. Daviaud E, Besada D, Leon N, Rohde S, Sanders D, Oliphant N, et al. Costs of implementing integrated community case management (iCCM) in six African countries: implications for sustainability. J Glob Health. 2017 Jun;7(1):010403. doi: 10.7189/jogh.07.010403

44. Escribano Ferrer B, Hansen KS, Gyapong M, Bruce J, Narh Bana SA, Narh CT, et al. Cost-effectiveness analysis of the national implementation of integrated community case management and community-based health planning and services in Ghana for the treatment of malaria, diarrhoea and pneumonia. Malar J. 2017 Jul 5;16(1):277. doi: 10.1186/s12936-017-1906-9

45. Kasteng F, Settumba S, Källander K, Vassall A, the inSCALE Study Group. Valuing the work of unpaid community health workers and exploring the incentives to volunteering in rural Africa. Health Policy Plan. 2016 Mar 1;31(2):205–16. doi: 10.1093/heapol/czv042

46. Nanyonjo A, Bagorogoza B, Kasteng F, Ayebale G, Makumbi F, Tomson G, et al. Estimating the cost of referral and willingness to pay for referral to higher-level health facilities: a case series study from an integrated community case management programme in Uganda. BMC Health Serv Res. 2015 Aug 28;15(1):347. doi: 10.1186/s12913-015-1019-5

47. Lubogo P, Lukyamuzi JE, Kyambadde D, Komakech AA, Kitutu FE, Mulogo EM. Cost-effectiveness analysis of integrated community case management delivery models utilizing drug sellers and community health workers for treatment of under-five febrile cases of malaria, pneumonia, diarrhoea in rural Uganda. Malar J. 2021 Oct 18;20(1):407. doi: 10.1186/s12936-021-03944-3

48. Machuki JA, Aduda DSO, Omondi AB, Onono MA. Patient-level cost of home- and facility-based child pneumonia treatment in Suba Sub County, Kenya. PLoS ONE. 2019 Nov 19;14(11):e0225194. doi: 10.1371/journal.pone.0225194

49. Niyibitegeka F, Riewpaiboon A, Sangroongruangsri S. Cost-effectiveness analysis of community case management of childhood diarrhea in Burundi. Value Health Reg Issues. 2021 Sep;25:157–64. doi: 10.1016/j.vhri.2021.03.005

50. Olsen M, Norheim OF, Memirie ST. Reducing regional health inequality: a sub-national distributional cost-effectiveness analysis

of community-based treatment of childhood pneumonia in Ethiopia. Int J Equity Health. 2021 Jan 6;20(1):9. doi: 10.1186/s12939-020-01328-8

51. Razakamanana MV, Audibert M, Andrianantoandro VT, Harimanana A. Impact and efficiency of integrating pneumonia diagnosis and treatment into malaria community case management in Madagascar. Rev Économique. 2020;71(1):5–30

52. Wagner Z, Zutshi R, Asiimwe JB, Levine D. The cost-effectiveness of community health workers delivering free diarrhoea treatment: evidence from Uganda. Health Policy Plan. 2022 Jan 13;37(1):123–31. doi: 10.1093/heapol/czab120

53. Mulogo E, Ntaro M, Wesuta A, Namusisi J, Kawungezi P, Batwala V, et al. Cost-effectiveness of village health worker-led integrated community case management (iCCM) versus health facility based management for childhood illnesses in rural southwestern Uganda. Malar J. 2024 May 15;23(1):147. doi: 10.1186/s12936-024-04962-7
