# Supplementary material for: Costs and cost-effectiveness of community health worker programs on reproductive, maternal, newborn and child health in low- and middle-income countries (2015–2024): A scoping review
Source: PLOS Glob Public Health. 2026 Jan 22;6(1):e0004893. doi: 10.1371/journal.pgph.0004893 (PMC12826514; doi:10.1371/journal.pgph.0004893)
Supplement: S1 Text — (PDF) [file pgph.0004893.s003.pdf]

Ovid MEDLINE(R) and Epub Ahead of Print, In-Process, In-Data-Review & Other Non-Indexed Citations and Daily (1946 to July 06, 2023)

1	volunteers/	10847
2	Hospital Volunteers/	1323
3	health auxiliar*.tw,kw.	46
4	peer group*.tw,kw.	3470
5	health visitor*.tw,kw.	2779
6	Doulas/	224
7	doula*.tw,kw.	535
8	douladural*.tw,kw.	1
9	((lay or voluntary or volunteer* or untrained or unlicensed or non-professional* or nonprofessional* or informal or non-formal) adj5 (worker* or visitor* or attendant* or aide* or support* or person* or helper* or carer* or caregiver* or care giver* or consultant* or assistant* or staff or visit* or midwife or midwives or provider* or practitioner*)).tw,kw.	22264
10	Allied Health Personnel/	13030
11	Community Health Workers/	6603
12	paramedics/	66
13	(paraprofessional* or paramedic* or allied health personnel or allied health worker* or support worker* or home health aide*).tw,kw.	13109
14	(trained adj3 (volunteer* or health worker* or mother*)).tw,kw.	2394
15	((communit* or village* or frontline*) adj3 (health worker* or health care worker* or healthcare worker* or distributor* or worker* or provider*)).tw,kw.	16975
16	(communit* adj3 (volunteer* or aide* or support)).tw,kw.	12108
17	((birth or childbirth or labor or labour) adj1 (attendant* or assistant*)).tw,kw.	2710
18	montrice*.tw,kw.	0

19	((lay or peer) adj1 (volunteer* or mentor* or counsel* or support or intervention*)).tw,kw.	9306
20	(church based adj3 (intervention* or program* or counsel*)).tw,kw.	196
21	(linkworker* or link worker*).tw,kw.	149
22	barefoot doctor*.tw,kw.	147
23	((health or healthcare) adj1 outreach).tw,kw.	535
24	Home Care Services/	36341
25	(home adj1 (care or aide* or nursing or support or intervention* or treatment* or visit*)).tw,kw.	60093
26	((care or aide* or nursing or support or intervention* or treatment* or visit*) adj3 (lay or volunteer* or voluntary)).tw,kw.	5985
27	(Auxiliary adj3 (worker* or nurse* or midwives or midwife)).tw,kw.	728
28	(expert patient* or health promoter* or health extension worker* or mentor mother*).tw,kw.	1619
29	or/1-28	185783
30	Economics/	27505
31	exp "Costs and Cost Analysis"/	265168
32	Economics, Nursing/	4013
33	Economics, Medical/	9249
34	exp "Fees and Charges"/	31382
35	exp Budgets/	14119
36	budget*.ti,ab,kf.	36039
37	(economic* or cost or costs or costly or costing or price or prices or pricing or expenditure or expenditures or expense or expenses or financial or finance or finances or financed).ti,kf.	279577
38	(economic* or cost or costs or costly or costing or price or prices or pricing or expenditure or expenditures or expense or expenses or financial or finance or finances or financed).ab. /freq=2	380289

39	(cost* adj2 (effective* or utilit* or benefit* or minimi* or analy* or outcome or outcomes)).ab,kf.	209257
40	(value adj2 (money or monetary)).ti,ab,kf.	3037
41	exp models, economic/	16224
42	economic model*.ab,kf.	4209
43	exp Decision Theory/	13301
44	(decision* adj2 (tree* or analy* or model*)).ti,ab,kf.	37770
45	Disability-Adjusted Life Years/	188
46	(Disability-Adjusted Life Years or DALYS).tw,kw.	4950
47	quality-adjusted life years/	15725
48	(quality-adjusted life years or QALYS).tw,kw.	13150
49	Return on investment.tw,kw.	2452
50	(multiple criteria decision analysis or MCDA).tw,kw.	972
51	Cost-Benefit Analysis/	92689
52	cost-benefit analysis.tw,kw.	5619
53	(Economic evaluation* or economic analys*).tw,kw.	23537
54	Financial management/	17074
55	(Financial planning or financial management).tw,kw.	1692
56	or/30-55	824735
58	29 and 56 and 57	3870
59	limit 58 to yr="2015 -Current"	1832

Embase Classic+Embase (1947 to 2023 July 07)

1	volunteer/	66214
2	Hospital Volunteer/	165
3	health auxiliar*.tw,kw.	71

4	peer group*.tw,kw.	4548
5	health visitor*.tw,kw.	3182
6	Doula/	367
7	doula*.tw,kw.	544
8	douladural*.tw,kw.	1
9	((lay or voluntary or volunteer* or untrained or unlicensed or non-professional* or nonprofessional* or informal or non-formal) adj5 (worker* or visitor* or attendant* or aide* or support* or person* or helper* or carer* or caregiver* or care giver* or consultant* or assistant* or staff or visit* or midwife or midwives or provider* or practitioner*)).tw,kw.	29630
10	paramedical personnel/	16681
11	health auxiliary/	9820
12	(paraprofessional* or paramedic* or allied health personnel or allied health worker* or support worker* or home health aide*).tw,kw.	19561
13	(trained adj3 (volunteer* or health worker* or mother*)).tw,kw.	3324
14	((communit* or village* or frontline*) adj3 (health worker* or health care worker* or healthcare worker* or distributor* or worker* or provider*)).tw,kw.	21315
15	(communit* adj3 (volunteer* or aide* or support)).tw,kw.	15857
16	((birth or childbirth or labor or labour) adj1 (attendant* or assistant*)).tw,kw.	2900
17	montrice*.tw,kw.	0
18	((lay or peer) adj1 (volunteer* or mentor* or counsel* or support or intervention*)).tw,kw.	12868
19	(church based adj3 (intervention* or program* or counsel*)).tw,kw.	230
20	(linkworker* or link worker*).tw,kw.	192
21	barefoot doctor*.tw,kw.	147
22	((health or healthcare) adj1 outreach).tw,kw.	725

23	Home Care/	72572
24	(home adj1 (care or aide* or nursing or support or intervention* or treatment* or visit*)).tw,kw.	79808
25	((care or aide* or nursing or support or intervention* or treatment* or visit*) adj3 (lay or volunteer* or voluntary)).tw,kw.	9150
26	(Auxiliary adj3 (worker* or nurse* or midwives or midwife)).tw,kw.	854
27	(expert patient* or health promoter* or health extension worker* or mentor mother*).tw,kw.	2112
28	or/1-27	310970
29	Economics/	247224
30	Cost/	66138
31	exp Health Economics/	1051128
32	exp Budgets/	34387
33	budget*.ti,ab,kf.	48974
34	(economic* or cost or costs or costly or costing or price or prices or pricing or expenditure or expenditures or expense or expenses or financial or finance or finances or financed).ti,kf.	355771
35	(economic* or cost or costs or costly or costing or price or prices or pricing or expenditure or expenditures or expense or expenses or financial or finance or finances or financed).ab. /freq=2	542091
36	(cost* adj2 (effective* or utilit* or benefit* or minimi* or analy* or outcome or outcomes)).ab,kf.	295373
37	(value adj2 (money or monetary)).ti,ab,kf.	4170
38	exp economic model/	3794
39	economic model*.ab,kf.	6345
40	Decision Theory/	1887
41	Decision Tree/	21876
42	(decision* adj2 (tree* or analy* or model*)).ti,ab,kf.	52577

43	Disability-Adjusted Life Year/	4550
44	(Disability-Adjusted Life Years or DALYS).tw,kw.	6446
45	quality-adjusted life year/	35690
46	(quality-adjusted life years or QALYS).tw,kw.	23305
47	Return on investment.tw,kw.	3276
48	(multiple criteria decision analysis or MCDA).tw,kw.	1534
49	cost benefit analysis/	94545
50	cost-benefit analysis.tw,kw.	8114
51	(Economic evaluation* or economic analys*).tw,kw.	33888
52	Financial management/	125641
53	(Financial planning or financial management).tw,kw.	2268
54	or/29-53	1768310

APA PsycInfo (1806 to July Week 1 2023)

1	volunteers/	6011
2	health auxiliar*.tw.	1
3	peer group*.tw.	6619
4	health visitor*.tw.	534
5	doula*.tw.	186
6	douladural*.tw.	0
7	((lay or voluntary or volunteer* or untrained or unlicensed or non-professional* or nonprofessional* or informal or non-formal) adj5 (worker* or visitor* or attendant* or aide* or support* or person* or helper* or carer* or caregiver* or care giver* or consultant* or assistant* or staff or visit* or midwife or midwives or provider* or practitioner*)).tw.	16010
8	Allied Health Personnel/	1351

9	paramedics/	440
10	(paraprofessional* or paramedic* or allied health personnel or allied health worker* or support worker* or home health aide*).tw.	5058
11	(trained adj3 (volunteer* or health worker* or mother*)).tw.	751
12	((communit* or village* or frontline*) adj3 (health worker* or health care worker* or healthcare worker* or distributor* or worker* or provider*)).tw.	6487
13	(communit* adj3 (volunteer* or aide* or support)).tw.	9618
14	((birth or childbirth or labor or labour) adj1 (attendant* or assistant*)).tw.	300
15	montrice*.tw.	0
16	((lay or peer) adj1 (volunteer* or mentor* or counsel* or support or intervention*)).tw.	8801
17	(church based adj3 (intervention* or program* or counsel*)).tw.	207
18	(linkworker* or link worker*).tw.	64
19	barefoot doctor*.tw.	8
20	((health or healthcare) adj1 outreach).tw.	282
21	Home Care/	7596
22	(home adj1 (care or aide* or nursing or support or intervention* or treatment* or visit*)).tw.	24703
23	((care or aide* or nursing or support or intervention* or treatment* or visit*) adj3 (lay or volunteer* or voluntary)).tw.	3081
24	(Auxiliary adj3 (worker* or nurse* or midwives or midwife)).tw.	131
25	(expert patient* or health promoter* or health extension worker* or mentor mother*).tw.	465
26	or/1-25	84206
27	Economics/	27668
28	exp "Costs and Cost Analysis"/	49131

29	Health Care Economics/	1183
30	exp Budgets/	1427
31	budget*.tw.	10217
32	(economic* or cost or costs or costly or costing or price or prices or pricing or expenditure or expenditures or expense or expenses or financial or finance or finances or financed).ti.	46803
33	(economic* or cost or costs or costly or costing or price or prices or pricing or expenditure or expenditures or expense or expenses or financial or finance or finances or financed).ab. /freq=2	105796
34	(cost* adj2 (effective* or utilit* or benefit* or minimi* or analy* or outcome or outcomes)).ab.	28207
35	(value adj2 (money or monetary)).tw.	1154
36	economic model*.ab.	1090
37	exp Decision Theory/	1852
38	(decision* adj2 (tree* or analy* or model*)).tw.	11140
39	(Disability-Adjusted Life Years or DALYS).tw.	539
40	(quality-adjusted life years or QALYS).tw.	1386
41	Return on investment.tw.	1050
42	(multiple criteria decision analysis or MCDA).tw.	125
43	cost-benefit analysis.tw.	1207
44	(Economic evaluation* or economic analys*).tw.	3545
45	(Financial planning or financial management).tw.	1195
46	or/27-45	184947

Global Health (1910 to 2023 Week 26)

1	Volunteers/	2600
2	health auxiliar*.ti,ab.	56

3	peer group*.ti,ab.	677
4	health visitor*.ti,ab.	1154
5	doula*.ti,ab.	81
6	douladural*.ti,ab.	0
7	((lay or voluntary or volunteer* or untrained or unlicensed or non-professional* or nonprofessional* or informal or non-formal) adj5 (worker* or visitor* or attendant* or aide* or support* or person* or helper* or carer* or caregiver* or care giver* or consultant* or assistant* or staff or visit* or midwife or midwives or provider* or practitioner*)).ti,ab.	5562
8	Community Health Services/	5894
9	(paraprofessional* or paramedic* or allied health personnel or allied health worker* or support worker* or home health aide*).ti,ab.	1994
10	(trained adj3 (volunteer* or health worker* or mother*)).ti,ab.	1133
11	((communit* or village* or frontline*) adj3 (health worker* or health care worker* or healthcare worker* or distributor* or worker* or provider*)).ti,ab.	8853
12	(communit* adj3 (volunteer* or aide* or support)).ti,ab.	4050
13	((birth or childbirth or labor or labour) adj1 (attendant* or assistant*)).ti,ab.	1780
14	montrice*.ti,ab.	0
15	((lay or peer) adj1 (volunteer* or mentor* or counsel* or support or intervention*)).ti,ab.	1930
16	(church based adj3 (intervention* or program* or counsel*)).ti,ab.	70
17	(linkworker* or link worker*).ti,ab.	55
18	barefoot doctor*.ti,ab.	70
19	((health or healthcare) adj1 outreach).ti,ab.	213
20	Home Care/	2644

21	(home adj1 (care or aide* or nursing or support or intervention* or treatment* or visit*)).ti,ab.	9497
22	((care or aide* or nursing or support or intervention* or treatment* or visit*) adj3 (lay or volunteer* or voluntary)).ti,ab.	1604
23	(Auxiliary adj3 (worker* or nurse* or midwives or midwife)).ti,ab.	417
24	(expert patient* or health promoter* or health extension worker* or mentor mother*).ti,ab.	979
25	or/1-24	41978
26	Economics/	17725
27	exp Costs/	37464
28	exp Economic Analysis/	20481
29	Budgets/	675
30	budget*.ti,ab.	8320
31	(economic* or cost or costs or costly or costing or price or prices or pricing or expenditure or expenditures or expense or expenses or financial or finance or finances or financed).ti.	50103
32	(economic* or cost or costs or costly or costing or price or prices or pricing or expenditure or expenditures or expense or expenses or financial or finance or finances or financed).ab. /freq=2	109726
33	(cost* adj2 (effective* or utilit* or benefit* or minimi* or analy* or outcome or outcomes)).ab.	40832
34	(value adj2 (money or monetary)).ti,ab.	988
35	economic model*.ab.	736
36	(decision* adj2 (tree* or analy* or model*)).ti,ab.	5165
37	(Disability-Adjusted Life Years or DALYS).ti,ab.	2998
38	(quality-adjusted life years or QALYS).ti,ab.	2117
39	Return on investment.ti,ab.	553
40	(multiple criteria decision analysis or MCDA).ti,ab.	233

41	cost-benefit analysis.ti,ab.	1198
42	(Economic evaluation* or economic analys*).ti,ab.	4589
43	(Financial planning or financial management).ti,ab.	299
44	or/26-43<br>(Afghanistan or Burundi or Burkina Faso or Central African Republic or Congo or Eritrea or Ethiopia or Guinea or Gambia or Guinea-Bissau or Liberia or Madagascar or Mali or Mozambique or Malawi or Niger or North Korea or Rwanda or Sudan or Sierra Leone or Somalia or South Sudan or Syrian Arab Republic or Chad or Togo or Uganda or Yemen or Zambia or Angola or Benin or Bangladesh or Bolivia or Bhutan or "cote d'ivoir*" or "cote d' ivoir*" or cote divoir* or cote d ivoir* or ivory coast* or Cameroon or Comoros or Cabo Verde or Djibouti or Algeria or Egypt or Micronesia or Ghana or Honduras or Haiti or Indonesia or India or Iran or Kenya or Kyrgyzstan or Cambodia or Kiribati or Laos or Lebanon or Sri Lanka or Lesotho or Morocco or Myanmar or Burma or Mongolia or Mauritania or Nigeria or Nicaragua or Nepal or Pakistan or Philippines or Papua New Guinea or "West Bank" or Gaza or Senegal or Solomon Islands or El Salvador or "Sao Tome and Principe" or Eswatini or Swaziland or Tajikistan or Timor-Leste or Tunisia or Tanzania or Ukraine or Uzbekistan or Vietnam or Vanuatu or Samoa or Zimbabwe or Afghan* or Burundian* or Burkinabe* or Central African* or Congolese or Eritrean* or Ethiopian* or Guinean* or Gambian* or Guinea-Bissauan* or Liberian* or Malagasy* or Malian* or Mozambican* or Malawian* or Nigerien* or North Korean* or Rwandan* or Sudanese or Sierra Leonean* or Somali* or Syrian or Chadian* or Togolese or Ugandan* or Yemeni* or Zambian* or Angolan* or Benin* or Bangladeshi* or Bolivian* or Bhutanese* or Ivorian* or Cameroonian* or Comorian* or Cape Verdean* or Djiboutian* or Algerian* or Egyptian* or Micronesian* or Ghanaian* or Honduran* or Haitian* or Indonesian* or (Indian* not indiana) or Iranian* or Kenyan* or Kyrgyz* or kirgiz* or kirghiz* or Cambodian* or Kiribatian* or Laotian* or Lebanese or Sri Lankan* or Basotho* or Moroccan* or Burmese or Mongolian* or Mauritanian* or Nigerian* or Nicaraguan* or Nepalese or Pakistani* or Filipino* or Papua New Guinean* or Palestinian* or Senegalese or Solomon Islander* or Salvadoran* or Salvadorian* or Sao Tomean* or Eswatini* or swazi* or swati* or Tajik* or Timorese or Tunisian* or Tanzanian* or Ukrainian* or Uzbek* or Vietnamese or Vanuatuan* or Samoan* or Zimbabwean* or africa* or arab* countr* or middle east* or global	173278

	south or sahara* or subsahara* or magreb* or maghrib* or west indies* or caribbean* or central america* or latin america* or south america* or central asia* or north asia* or northern asia* or southeastern asia* or south eastern asia* or southeast asia* or south east asia* or west asia* or western asia* or east europe* or eastern europe* or developing countr* or developing nation* or developing population* or developing world or less developed countr* or less developed nation* or less developed world or lesser developed countr* or lesser developed nation* or lesser developed world or under developed countr* or under developed nation* or under developed world or underdeveloped countr* or underdeveloped nation* or underdeveloped world or middle income countr* or middle income nation* or middle income population* or low income countr* or low income nation* or low income population* or lower income countr* or lower income nation* or lower income population* or underserved countr* or underserved nation* or underserved population* or under served population* or under served nation* or under served population* or deprived countr* or deprived population* or high burden countr* or high burden nation* or countdown countr* or countdown nation* or poor countr* or poor nation* or poor population* or poor world or poorer countr* or poorer nation* or poorer population* or poorer world or developing econom* or less developed econom* or underdeveloped econom* or under developed econom* or middle income econom* or low income econom* or lower income econom* or low gdp or low gnp or low gross domestic or low gross national or lower gdp or lower gnp or lower gross domestic or lower gross national or lmic or lmics or third world or lami countr* or transitional countr* or emerging econom* or emerging nation*).ti,ab,hw.	
46	25 and 44 and 45	2661
47	limit 46 to yr="2015 -Current"	1434

AMED (Allied and Complementary Medicine) (1985 to June 2023)

1	Voluntary Workers/	206
2	Hospital Volunteers/	13
3	health auxiliar*.ti,ab.	0
4	peer group*.ti,ab.	56

5	health visitor*.ti,ab.	29
6	doula*.ti,ab.	7
7	douladural*.ti,ab.	0
8	((lay or voluntary or volunteer* or untrained or unlicensed or non-professional* or nonprofessional* or informal or non-formal) adj5 (worker* or visitor* or attendant* or aide* or support* or person* or helper* or carer* or caregiver* or care giver* or consultant* or assistant* or staff or visit* or midwife or midwives or provider* or practitioner*)).ti,ab.	1017
9	Allied Health Personnel/	691
10	Community Health Services/	2165
11	(paraprofessional* or paramedic* or allied health personnel or allied health worker* or support worker* or home health aide*).ti,ab.	258
12	(trained adj3 (volunteer* or health worker* or mother*)).ti,ab.	49
13	((communit* or village* or frontline*) adj3 (health worker* or health care worker* or healthcare worker* or distributor* or worker* or provider*)).ti,ab.	242
14	(communit* adj3 (volunteer* or aide* or support)).ti,ab.	553
15	((birth or childbirth or labor or labour) adj1 (attendant* or assistant*)).ti,ab.	6
16	montrice*.ti,ab.	0
17	((lay or peer) adj1 (volunteer* or mentor* or counsel* or support or intervention*)).ti,ab.	348
18	(church based adj3 (intervention* or program* or counsel*)).ti,ab.	3
19	(linkworker* or link worker*).ti,ab.	2
20	barefoot doctor*.ti,ab.	3
21	((health or healthcare) adj1 outreach).ti,ab.	9
22	Home Care Services/	1994

23	(home adj1 (care or aide* or nursing or support or intervention* or treatment* or visit*)).ti,ab.	2889
24	((care or aide* or nursing or support or intervention* or treatment* or visit*) adj3 (lay or volunteer* or voluntary)).ti,ab.	468
25	(Auxiliary adj3 (worker* or nurse* or midwives or midwife)).ti,ab.	5
26	(expert patient* or health promoter* or health extension worker* or mentor mother*).ti,ab.	27
27	or/1-26	9229
28	Economics/	4859
29	exp "Costs and Cost Analysis"/	1560
30	budget*.ti,ab.	223
31	(economic* or cost or costs or costly or costing or price or prices or pricing or expenditure or expenditures or expense or expenses or financial or finance or finances or financed).ti.	2504
32	(economic* or cost or costs or costly or costing or price or prices or pricing or expenditure or expenditures or expense or expenses or financial or finance or finances or financed).ab. /freq=2	3305
33	(cost* adj2 (effective* or utilit* or benefit* or minimi* or analy* or outcome or outcomes)).ab.	2076
34	(value adj2 (money or monetary)).ti,ab.	21
35	economic model*.ab.	7
36	(decision* adj2 (tree* or analy* or model*)).ti,ab.	260
37	(Disability-Adjusted Life Years or DALYS).ti,ab.	19
38	(quality-adjusted life years or QALYS).ti,ab.	109
39	Return on investment.ti,ab.	13
40	(multiple criteria decision analysis or MCDA).ti,ab.	1
41	Cost-Benefit Analysis/	698
42	cost-benefit analysis.ti,ab.	48

43	(Economic evaluation* or economic analys*).ti,ab.	214
44	Financial management/	132
45	(Financial planning or financial management).ti,ab.	42
46	or/28-45<br>Afghanistan or Burundi or Burkina Faso or Central African Republic or Congo or Eritrea or Ethiopia or Guinea or Gambia or Guinea-Bissau or Liberia or Madagascar or Mali or Mozambique or Malawi or Niger or North Korea or Rwanda or Sudan or Sierra Leone or Somalia or South Sudan or Syrian Arab Republic or Chad or Togo or Uganda or Yemen or Zambia or Angola or Benin or Bangladesh or Bolivia or Bhutan or "cote d'ivoir*" or "cote d' ivoir*" or cote divoir* or cote d ivoir* or ivory coast* or Cameroon or Comoros or Cabo Verde or Djibouti or Algeria or Egypt or Micronesia or Ghana or Honduras or Haiti or Indonesia or India or Iran or Kenya or Kyrgyzstan or Cambodia or Kiribati or Laos or Lebanon or Sri Lanka or Lesotho or Morocco or Myanmar or Burma or Mongolia or Mauritania or Nigeria or Nicaragua or Nepal or Pakistan or Philippines or Papua New Guinea or "West Bank" or Gaza or Senegal or Solomon Islands or El Salvador or "Sao Tome and Principe" or Eswatini or Swaziland or Tajikistan or Timor-Leste or Tunisia or Tanzania or Ukraine or Uzbekistan or Vietnam or Vanuatu or Samoa or Zimbabwe or Afghan* or Burundian* or Burkinabe* or Central African* or Congolese or Eritrean* or Ethiopian* or Guinean* or Gambian* or Guinea-Bissauan* or Liberian* or Malagasy* or Malian* or Mozambican* or Malawian* or Nigerien* or North Korean* or Rwandan* or Sudanese or Sierra Leonean* or Somali* or Syrian or Chadian* or Togolese or Ugandan* or Yemeni* or Zambian* or Angolan* or Benin* or Bangladeshi* or Bolivian* or Bhutanese* or Ivorian* or Cameroonian* or Comorian* or Cape Verdean* or Djiboutian* or Algerian* or Egyptian* or Micronesian* or Ghanaian* or Honduran* or Haitian* or Indonesian* or (Indian* not indiana) or Iranian* or Kenyan* or Kyrgyz* or kirgiz* or kirghiz* or Cambodian* or Kiribatian* or Laotian* or Lebanese or Sri Lankan* or Basotho* or Moroccan* or Burmese or Mongolian* or Mauritanian* or Nigerian* or Nicaraguan* or Nepalese or Pakistani* or Filipino* or Papua New Guinean* or Palestinian* or Senegalese or Solomon Islander* or Salvadoran* or Salvadorian* or Sao Tomean* or Eswatini* or swazi* or swati* or Tajik* or Timorese or Tunisian* or Tanzanian* or Ukrainian* or Uzbek* or Vietnamese or Vanuatuan* or Samoan* or Zimbabwean* or africa* or arab* countr* or middle east* or global south or sahara* or subsahara* or magreb* or maghrib* or west indies* or caribbean* or central america* or latin america* or south america* or	9614

	central asia* or north asia* or northern asia* or southeastern asia* or south eastern asia* or southeast asia* or south east asia* or west asia* or western asia* or east europe* or eastern europe* or developing countr* or developing nation* or developing population* or developing world or less developed countr* or less developed nation* or less developed world or lesser developed countr* or lesser developed nation* or lesser developed world or under developed countr* or under developed nation* or under developed world or underdeveloped countr* or underdeveloped nation* or underdeveloped world or middle income countr* or middle income nation* or middle income population* or low income countr* or low income nation* or low income population* or lower income countr* or lower income nation* or lower income population* or underserved countr* or underserved nation* or underserved population* or under served population* or under served nation* or under served population* or deprived countr* or deprived population* or high burden countr* or high burden nation* or countdown countr* or countdown nation* or poor countr* or poor nation* or poor population* or poor world or poorer countr* or poorer nation* or poorer population* or poorer world or developing econom* or less developed econom* or underdeveloped econom* or under developed econom* or middle income econom* or low income econom* or lower income econom* or low gdp or low gnp or low gross domestic or low gross national or lower gdp or lower gnp or lower gross domestic or lower gross national or lmic or lmics or third world or lami countr* or transitional countr* or emerging econom* or emerging nation*).ti,ab,hw.	
48	27 and 46 and 47	39
49	limit 48 to yr="2015 -Current"	14

Cochrane Central Register of Controlled Trials

ID	Search	Hits
#1	MeSH descriptor: [Volunteers] this term only	382
#2	MeSH descriptor: [Hospital Volunteers] this term only	3
#3	(health auxiliar*):ti,ab,kw	1044
#4	(peer group*):ti,ab,kw	9120
#5	(health visitor*):ti,ab,kw	606

#6	MeSH descriptor: [Doulas] this term only	12
#7	(doula* OR douladural*):ti,ab,kw	94
#8	((lay OR voluntary OR volunteer* OR untrained OR unlicensed OR non-professional* OR nonprofessional* OR informal OR non-formal) NEAR/5 (worker* OR visitor* OR attendant* OR aide* OR support* OR person* OR helper* OR carer* OR caregiver* OR care giver* OR consultant* OR assistant* OR staff OR visit* OR midwife OR midwives OR provider* OR practitioner*)):ti,ab,kw	4590
#9	MeSH descriptor: [Allied Health Personnel] this term only	343
#10	MeSH descriptor: [Community Health Workers] this term only	706
#11	MeSH descriptor: [Paramedics] this term only	3
#12	(paraprofessional* OR paramedic* OR allied health personnel OR allied health worker* OR support worker* OR home health aide*):ti,ab,kw	5104
#13	(trained NEAR/3 (volunteer* OR health worker* OR mother*)):ti,ab,kw	1916
#14	((communit* OR village* OR frontline*) NEAR/3 (health worker* OR health care worker* OR healthcare worker* OR distributor* OR worker* OR provider*)):ti,ab,kw	13934
#15	(communit* NEAR/3 (volunteer* OR aide* OR support)):ti,ab,kw	1540
#16	((birth OR childbirth OR labor OR labour) NEAR/1 (attendant* OR assistant*)):ti,ab,kw	289
#17	(montrice*):ti,ab,kw	0
#18	((lay OR peer) NEAR/1 (volunteer* OR mentor* OR counsel* OR support OR intervention*)):ti,ab,kw	2622
#19	(church based NEAR/3 (intervention* OR program* OR counsel*)):ti,ab,kw	174
#20	(linkworker* OR link worker*):ti,ab,kw	262
#21	(barefoot doctor*):ti,ab,kw	4
#22	((health OR healthcare) NEAR/1 outreach):ti,ab,kw	58
#23	MeSH descriptor: [Home Care Services] this term only	2155

#24	(home NEAR/1 (care OR aide* OR nursing OR support OR intervention* OR treatment* OR visit*)):ti,ab,kw	15859
#25	((care OR aide* OR nursing OR support OR intervention* OR treatment* OR visit*) NEAR/3 (lay OR volunteer* OR voluntary)):ti,ab,kw	3363
#26	(Auxiliary NEAR/3 (worker* OR nurse* OR midwives OR midwife)):ti,ab,kw	62
#27	(expert patient* OR health promoter* OR health extension worker* OR mentor mother*):ti,ab,kw	7172
#28	{OR #1-#27}	55315
#29	MeSH descriptor: [Economics] this term only	84
#30	MeSH descriptor: [Costs and Cost Analysis] explode all trees	14572
#31	MeSH descriptor: [Economics, Nursing] this term only	13
#32	MeSH descriptor: [Economics, Medical] this term only	32
#33	MeSH descriptor: [Fees and Charges] explode all trees	321
#34	MeSH descriptor: [Budgets] explode all trees	56
#35	(budget*):ti,ab,kw	1466
#36	(economic* OR cost OR costs OR costly OR costing OR price OR prices OR pricing OR expenditure OR expenditures OR expense OR expenses OR financial OR finance OR finances OR financed):ti,kw	44528
#37	(cost* NEAR/2 (effective* OR utilit* OR benefit* OR minimi* OR analy* OR outcome OR outcomes)):ab,kw	40612
#38	(value NEAR/2 (money OR monetary)):ti,ab,kw	369
#39	MeSH descriptor: [Models, Economic] explode all trees	571
#40	(economic model*):ti,ab,kw	4999
#41	MeSH descriptor: [Decision Theory] explode all trees	339
#42	(decision* NEAR/2 (tree* OR analy* OR model*)):ti,ab,kw	3202
#43	MeSH descriptor: [Disability-Adjusted Life Years] this term only	3

#44	(Disability-Adjusted Life Years OR DALYS):ti,ab,kw	279
#45	MeSH descriptor: [Quality-Adjusted Life Years] this term only	1940
#46	(quality-adjusted life years OR QALYS):ti,ab,kw	5866
#47	(Return on investment):ti,ab,kw	223
#48	(multiple criteria decision analysis OR MCDA):ti,ab,kw	462
#49	MeSH descriptor: [Cost-Benefit Analysis] this term only	9824
#50	(cost-benefit analysis):ti,ab,kw	12281
#51	(Economic evaluation* OR economic analys*):ti,ab,kw	14683
#52	MeSH descriptor: [Financial Management] this term only	95
#53	(Financial planning OR financial management):ti,ab,kw	2613
#54	{OR #29-#53}<br>(Afghanistan OR Burundi OR Burkina Faso OR Central African Republic OR Congo OR Eritrea OR Ethiopia OR Guinea OR Gambia OR Guinea-Bissau OR Liberia OR Madagascar OR Mali OR Mozambique OR Malawi OR Niger OR North Korea OR Rwanda OR Sudan OR Sierra Leone OR Somalia OR South Sudan OR Syrian Arab Republic OR Chad OR Togo OR Uganda OR Yemen OR Zambia OR Angola OR Benin OR Bangladesh OR Bolivia OR Bhutan OR cote d'ivoir* OR cote d' ivoir* OR cote divoir* OR cote d ivoir* OR ivory coast* OR Cameroon OR Comoros OR Cabo Verde OR Djibouti OR Algeria OR Egypt OR Micronesia OR Ghana OR Honduras OR Haiti OR Indonesia OR India OR Iran OR Kenya OR Kyrgyzstan OR Cambodia OR Kiribati OR Laos OR Lebanon OR Sri Lanka OR Lesotho OR Morocco OR Myanmar OR Burma OR Mongolia OR Mauritania OR Nigeria OR Nicaragua OR Nepal OR Pakistan OR Philippines OR Papua New Guinea OR "West Bank" OR Gaza OR Senegal OR Solomon Islands OR El Salvador OR "Sao Tome and Principe" OR Eswatini OR Swaziland OR Tajikistan OR Timor-Leste OR Tunisia OR Tanzania OR Ukraine OR Uzbekistan OR Vietnam OR Vanuatu OR Samoa OR Zimbabwe OR Afghan* OR Burundian* OR Burkinabe* OR Central African* OR Congolese OR Eritrean* OR Ethiopian* OR Guinean* OR Gambian* OR Guinea-Bissauan* OR Liberian* OR Malagasy* OR Malian* OR Mozambican* OR Malawian* OR Nigerien* OR North Korean* OR Rwandan* OR Sudanese OR Sierra Leonean* OR Somali* OR Syrian OR Chadian* OR Togolese OR Ugandan* OR Yemeni* OR Zambian* OR Angolan*	70292

	OR Benin* OR Bangladeshi* OR Bolivian* OR Bhutanese* OR Ivorian* OR Cameroonian* OR Comorian* OR Cape Verdean* OR Djiboutian* OR Algerian* OR Egyptian* OR Micronesian* OR Ghanaian* OR Honduran* OR Haitian* OR Indonesian* OR (Indian* not indiana) OR Iranian* OR Kenyan* OR Kyrgyz* OR kirgiz* OR kirghiz* OR Cambodian* OR Kiribatian* OR Laotian* OR Lebanese OR Sri Lankan* OR Basotho* OR Moroccan* OR Burmese OR Mongolian* OR Mauritanian* OR Nigerian* OR Nicaraguan* OR Nepalese OR Pakistani* OR Filipino* OR Papua New Guinean* OR Palestinian* OR Senegalese OR Solomon Islander* OR Salvadoran* OR Salvadorian* OR Sao Tomean* OR Eswatini* OR swazi* OR swati* OR Tajik* OR Timorese OR Tunisian* OR Tanzanian* OR Ukrainian* OR Uzbek* OR Vietnamese OR Vanuatuan* OR Samoan* OR Zimbabwean* OR africa* OR arab* countr* OR middle east* OR global south OR sahara* OR subsahara* OR magreb* OR maghrib* OR west indies* OR caribbean* OR central america* OR latin america* OR south america* OR central asia* OR north asia* OR northern asia* OR southeastern asia* OR south eastern asia* OR southeast asia* OR south east asia* OR west asia* OR western asia* OR east europe* OR eastern europe* OR developing countr* OR developing nation* OR developing population* OR developing world OR less developed countr* OR less developed nation* OR less developed world OR lesser developed countr* OR lesser developed nation* OR lesser developed world OR under developed countr* OR under developed nation* OR under developed world OR underdeveloped countr* OR underdeveloped nation* OR underdeveloped world OR middle income countr* OR middle income nation* OR middle income population* OR low income countr* OR low income nation* OR low income population* OR lower income countr* OR lower income nation* OR lower income population* OR underserved countr* OR underserved nation* OR underserved population* OR under served population* OR under served nation* OR under served population* OR deprived countr* OR deprived population* OR high burden countr* OR high burden nation* OR countdown countr* OR countdown nation* OR poor countr* OR poor nation* OR poor population* OR poor world OR poorer countr* OR poorer nation* OR poorer population* OR poorer world OR developing econom* OR less developed econom* OR underdeveloped econom* OR under developed econom* OR middle income econom* OR low income econom* OR lower income econom* OR low gdp OR low gnp OR low gross domestic OR low gross national OR lower gdp OR lower gnp OR lower gross domestic OR lower gross national OR lmic OR lmics	

	OR third world OR lami countr* OR transitional countr* OR emerging econom* OR emerging nation*):ti,ab,kw	
#56	#28 AND #54 AND #55 with Publication Year from 2015 to 2023, in Trials	1550

CINAHL

S1	MH Volunteers OR MH Hospital Volunteers OR TI (“Health auxiliar*” OR “peer group*” OR “health visitor*” OR doula* OR “Allied health personnel” OR “Community health worker*” OR paramedic* OR paraprofessional* “allied health worker*” OR “support worker*” OR “support worker*” OR “home health aide*” OR montrice* OR linkworker* OR “link worker*” OR “barefoot doctor*” OR “expert patient*” OR “health promoter*” OR “health extension worker*” OR “mentor mother*”) OR AB (“Health auxiliar*” OR “peer group*” OR “health visitor*” OR doula* OR “Allied health personnel” OR “Community health worker*” OR paramedic* OR paraprofessional* “allied health worker*” OR “support worker*” OR “support worker*” OR “home health aide*” OR montrice* OR linkworker* OR “link worker*” OR “barefoot doctor*” OR “expert patient*” OR “health promoter*” OR “health extension worker*” OR “mentor mother*”) OR TI ((lay OR voluntary OR volunteer* OR untrained OR unlicensed OR non-professional* OR nonprofessional* OR informal OR non-formal) N5 (worker* OR visitor* OR attendant* OR aide* OR support* OR person* OR helper* OR carer* OR caregiver* OR care giver* OR consultant* OR assistant* OR staff OR visit* OR midwife OR midwives OR provider* OR practitioner*)) OR AB ((lay OR voluntary OR volunteer* OR untrained OR unlicensed OR non-professional* OR nonprofessional* OR informal OR non-formal) N5 (worker* OR visitor* OR attendant* OR aide* OR support* OR person* OR helper* OR carer* OR caregiver* OR care giver* OR consultant* OR assistant* OR staff OR visit* OR midwife OR midwives OR provider* OR practitioner*)) OR TI (trained N3 (volunteer* OR “health worker*” OR mother*)) OR AB (trained N3 (volunteer* OR “health worker*” OR mother*)) OR TI ((communit* OR village* OR frontline*) N3 (“health worker*” OR “health care worker*” OR “healthcare worker*” OR distributor* OR worker* OR provider*)) OR AB ((communit* OR village* OR frontline*) N3 (“health worker*” OR “health care worker*” OR “healthcare worker*” OR distributor* OR worker* OR provider*)) OR TI (communit* N3 (volunteer* OR aide* OR support)) OR AB (communit* N3 (volunteer* OR aide* OR support))	51,954

S1	MH Volunteers OR MH Hospital Volunteers OR TI (“Health auxiliar*” OR “peer group*” OR “health visitor*” OR doula* OR “Allied health personnel” OR “Community health worker*” OR paramedic* OR paraprofessional* “allied health worker*” OR “support worker*” OR “support worker*” OR “home health aide*” OR montrice* OR linkworker* OR “link worker*” OR “barefoot doctor*” OR “expert patient*” OR “health promoter*” OR “health extension worker*” OR “mentor mother*”) OR AB (“Health auxiliar*” OR “peer group*” OR “health visitor*” OR doula* OR “Allied health personnel” OR “Community health worker*” OR paramedic* OR paraprofessional* “allied health worker*” OR “support worker*” OR “support worker*” OR “home health aide*” OR montrice* OR linkworker* OR “link worker*” OR “barefoot doctor*” OR “expert patient*” OR “health promoter*” OR “health extension worker*” OR “mentor mother*”) OR TI ((lay OR voluntary OR volunteer* OR untrained OR unlicensed OR non-professional* OR nonprofessional* OR informal OR non-formal) N5 (worker* OR visitor* OR attendant* OR aide* OR support* OR person* OR helper* OR carer* OR caregiver* OR care giver* OR consultant* OR assistant* OR staff OR visit* OR midwife OR midwives OR provider* OR practitioner*)) OR AB ((lay OR voluntary OR volunteer* OR untrained OR unlicensed OR non-professional* OR nonprofessional* OR informal OR non-formal) N5 (worker* OR visitor* OR attendant* OR aide* OR support* OR person* OR helper* OR carer* OR caregiver* OR care giver* OR consultant* OR assistant* OR staff OR visit* OR midwife OR midwives OR provider* OR practitioner*)) OR TI (trained N3 (volunteer* OR “health worker*” OR mother*)) OR AB (trained N3 (volunteer* OR “health worker*” OR mother*)) OR TI ((communit* OR village* OR frontline*) N3 (“health worker*” OR “health care worker*” OR “healthcare worker*” OR distributor* OR worker* OR provider*)) OR AB ((communit* OR village* OR frontline*) N3 (“health worker*” OR “health care worker*” OR “healthcare worker*” OR distributor* OR worker* OR provider*)) OR TI (communit* N3 (volunteer* OR aide* OR support)) OR AB (communit* N3 (volunteer* OR aide* OR support))	51,954

S2	TI ((birth OR childbirth OR labor OR labour) N1 (attendant* OR assistant*)) OR AB ((birth OR childbirth OR labor OR labour) N1 (attendant* OR assistant*)) OR TI ((lay OR peer) N1 (volunteer* OR mentor* OR counsel* OR support OR intervention*)) OR AB ((lay OR peer) N1 (volunteer* OR mentor* OR counsel* OR support OR intervention*)) OR TI (("church based" N3 (intervention* OR program* OR counsel*))) OR AB (("church based" N3 (intervention* OR program* OR counsel*))) OR TI (((health OR healthcare) N1 outreach)) OR AB (((health OR healthcare) N1 outreach)) OR TI ((home N1 (care OR aide* OR nursing OR support OR intervention* OR treatment* OR visit*))) OR AB ((home N1 (care OR aide* OR nursing OR support OR intervention* OR treatment* OR visit*))) OR TI (((care OR aide* OR nursing OR support OR intervention* OR treatment* OR visit*) N3 (lay OR volunteer* OR voluntary))) OR AB (((care OR aide* OR nursing OR support OR intervention* OR treatment* OR visit*) N3 (lay OR volunteer* OR voluntary)))	78,769
S3	TI ((Auxiliary N3 (worker* OR nurse* OR midwives OR midwife))) AND AB ((Auxiliary N3 (worker* OR nurse* OR midwives OR midwife))) OR MH Allied Health Personnel OR MH community Health Workers OR MH paramedics OR MH Home Care Services	9,406
S4	S1 OR S2 OR S3	129,588

S5	MH Economics OR MH (Costs and Cost Analysis) OR MH Economics, Nursing OR MH Economics, Medical OR MH (Fees and Charges) OR MH Budgets OR TI (economic* OR cost OR costs OR costly OR costing OR price OR prices OR pricing OR expenditure OR expenditures OR expense OR expenses OR financial OR finance OR finances OR financed) OR AB (cost* N2 (effective* OR utilit* OR benefit* OR minimi* OR analy* OR outcome OR outcomes)) OR TI ((value N2 (money OR monetary)) OR (“economic model*”) OR (decision* N2 (tree* OR analy* OR model*))) OR AB ((value N2 (money OR monetary)) OR (“economic model*”) OR (decision* N2 (tree* OR analy* OR model*))) OR TI (("Disability-Adjusted Life Years" OR DALYS OR "quality-adjusted life years" OR QALYS OR “Return on investment” OR “multiple criteria decision analysis” or MCDA OR “cost-benefit analysis” OR “Economic evaluation*” OR “economic analys*” OR “Financial planning” OR “financial management”)) OR AB (("Disability-Adjusted Life Years" OR DALYS OR "quality-adjusted life years" OR QALYS OR “Return on investment” OR “multiple criteria decision analysis” or MCDA OR “cost-benefit analysis” OR “Economic evaluation*” OR “economic analys*” OR “Financial planning” OR “financial management”))	177,125

	R Kyrgyz* OR kirgiz* OR kirghiz* OR Cambodian* OR Kiribatian* OR Laotian* OR Lebanese OR "Sri Lankan*" OR Basotho* OR Moroccan* OR Burmese OR Mongolian* OR Mauritanian* OR Nigerian* OR Nicaraguan* OR Nepalese OR Pakistani* OR Filipino* OR "Papua New Guinean*" OR Palestinian* OR Senegalese OR "Solomon Islander*" OR Salvadoran* OR Salvadorian* OR Sao Tomean* OR Eswatini* OR swazi* OR swati* OR Tajik* OR Timorese OR Tunisian* OR Tanzanian* OR Ukrainian* OR Uzbek* OR Vietnamese OR Vanuatuan* OR Samoan* OR Zimbabwean* OR africa* OR "arab* countr*" OR "middle east*" OR "global south" OR sahara* OR subsahara* OR magreb* OR maghrib* OR "west indies*" OR caribbean* OR "central america*" OR "latin america*" OR "south america*" OR "central asia*" OR "north asia*" OR "northern asia*" OR "southeastern asia*" OR "south eastern asia*" OR "southeast asia*" OR "south east asia*" OR "west asia*" OR "western asia*" OR "east europe*" OR "eastern europe*" OR "developing countr*" OR "developing nation*" OR "developing population*" OR "developing world" OR "less developed countr*" OR "less developed nation*" OR "less developed world" OR "lesser developed countr*" OR "lesser developed nation*" OR "lesser developed world" OR "under developed countr*" OR "under developed nation*" OR "under developed world" OR "underdeveloped countr*" OR "underdeveloped nation*" OR "underdeveloped world" OR "middle income countr*" OR "middle income nation*" OR "middle income population*" OR "low income countr*" OR "low income nation*" OR "low income population*" OR "lower income countr*" OR "lower income nation*" OR "lower income population*" OR "underserved countr*" OR "underserved nation*" OR "underserved population*" OR "under served population*" OR "under served nation*" OR "deprived countr*" OR "deprived population*" OR "high burden countr*" OR "high burden nation*" OR "countdown countr*" OR "countdown nation*" OR "poor countr*" OR "poor nation*" OR "poor population*" OR "poor world" OR "poorer countr*" OR "poorer nation*" OR "poorer population*" OR "poorer world" OR "developing econom*" OR "less developed econom*" OR "underdeveloped econom*" OR "under developed econom*" OR "middle income econom*" OR "low income econom*" OR "lower income econom*" OR "low gdp" OR "low gnp" OR "low gross domestic" OR "low gross national" OR "lower gdp" OR "lower gnp" OR "lower gross domestic" OR "lower gross national" OR lmic OR lmics OR "third world" OR "lami countr*" OR "transitional countr*" OR "emerging econom*" OR "emerging nation*")) OR AB ((Afghanistan OR Burundi OR "Burkina Faso" OR "Central African Republic" OR Congo OR Eritrea OR	

	Ethiopia OR Guinea OR Gambia OR Guinea-Bissau OR Liberia OR Madagascar OR Mali OR Mozambique OR Malawi OR Niger OR “North Korea” OR Rwanda OR Sudan OR “Sierra Leone” OR Somalia OR “South Sudan” OR “Syrian Arab Republic” OR Chad OR Togo OR Uganda OR Yemen OR Zambia OR Angola OR Benin OR Bangladesh OR Bolivia OR Bhutan OR "cote d'ivoir*" OR "cote d' ivoir*" OR “cote divoir*” OR “cote d ivoir*” OR “ivory coast*” OR Cameroon OR Comoros OR “Cabo Verde” OR Djibouti OR Algeria OR Egypt OR Micronesia OR Ghana OR Honduras OR Haiti OR Indonesia OR India OR Iran OR Kenya OR Kyrgyzstan OR Cambodia OR Kiribati OR Laos OR Lebanon OR “Sri Lanka” OR Lesotho OR Morocco OR Myanmar OR Burma OR Mongolia OR Mauritania OR Nigeria OR Nicaragua OR Nepal OR Pakistan OR Philippines OR “Papua New Guinea” OR "West Bank" OR Gaza OR Senegal OR “Solomon Islands” OR “El Salvador” OR "Sao Tome and Principe" OR Eswatini OR Swaziland OR Tajikistan OR Timor-Leste OR Tunisia OR Tanzania OR Ukraine OR Uzbekistan OR Vietnam OR Vanuatu OR Samoa OR Zimbabwe OR Afghan* OR Burundian* OR Burkinabe* OR “Central African*” OR Congolese OR Eritrean* OR Ethiopian* OR Guinean* OR Gambian* OR Guinea-Bissauan* OR Liberian* OR Malagasy* OR Malian* OR Mozambican* OR Malawian* OR Nigerien* OR “North Korean*” OR Rwandan* OR Sudanese OR “Sierra Leonean*” OR Somali* OR Syrian OR Chadian* OR Togolese OR Ugandan* OR Yemeni* OR Zambian* OR Angolan* OR Benin* OR Bangladeshi* OR Bolivian* OR Bhutanese* OR Ivorian* OR Cameroonian* OR Comorian* OR Cape Verdean* OR Djiboutian* OR Algerian* OR Egyptian* OR Micronesian* OR Ghanaian* OR Honduran* OR Haitian* OR Indonesian* OR (Indian* NOT indiana) OR Iranian* OR Kenyan* OR Kyrgyz* OR kirgiz* OR kirghiz* OR Cambodian* OR Kiribatian* OR Laotian* OR Lebanese OR “Sri Lankan*” OR Basotho* OR Moroccan* OR Burmese OR Mongolian* OR Mauritanian* OR Nigerian* OR Nicaraguan* OR Nepalese OR Pakistani* OR Filipino* OR “Papua New Guinean*” OR Palestinian* OR Senegalese OR “Solomon Islander*” OR Salvadoran* OR Salvadorian* OR Sao Tomean* OR Eswatini* OR swazi* OR swati* OR Tajik* OR Timorese OR Tunisian* OR Tanzanian* OR Ukrainian* OR Uzbek* OR Vietnamese OR Vanuatuan* OR Samoan* OR Zimbabwean* OR africa* OR “arab* countr*” OR “middle east*” OR “global south” OR sahara* OR subsahara* OR magreb* OR maghrib* OR “west indies*” OR caribbean* OR “central america*” OR “latin america*” OR “south america*” OR “central asia*” OR “north asia*” OR “northern asia*” OR “southeastern asia*” OR “south eastern asia*” OR “southeast asia*” OR “south east	

	asia*" OR "west asia*" OR "western asia*" OR "east europe*" OR "eastern europe*" OR "developing countr*" OR "developing nation*" OR "developing population*" OR "developing world" OR "less developed countr*" OR "less developed nation*" OR "less developed world" OR "lesser developed countr*" OR "lesser developed nation*" OR "lesser developed world" OR "under developed countr*" OR "under developed nation*" OR "under developed world" OR "underdeveloped countr*" OR "underdeveloped nation*" OR "underdeveloped world" OR "middle income countr*" OR "middle income nation*" OR "middle income population*" OR "low income countr*" OR "low income nation*" OR "low income population*" OR "lower income countr*" OR "lower income nation*" OR "lower income population*" OR "underserved countr*" OR "underserved nation*" OR "underserved population*" OR "under served population*" OR "under served nation*" OR "deprived countr*" OR "deprived population*" OR "high burden countr*" OR "high burden nation*" OR "countdown countr*" OR "countdown nation*" OR "poor countr*" OR "poor nation*" OR "poor population*" OR "poor world" OR "poorer countr*" OR "poorer nation*" OR "poorer population*" OR "poorer world" OR "developing econom*" OR "less developed econom*" OR "underdeveloped econom*" OR "under developed econom*" OR "middle income econom*" OR "low income econom*" OR "lower income econom*" OR "low gdp" OR "low gnp" OR "low gross domestic" OR "low gross national" OR "lower gdp" OR "lower gnp" OR "lower gross domestic" OR "lower gross national" OR lmic OR lmics OR "third world" OR "lami countr*" OR "transitional countr*" OR "emerging econom*" OR "emerging nation*"))	
S7	S4 AND S5 AND S6 Limiters - Published Date: 20150101-20231231<br><br>Search modes - Boolean/Phrase	309

Web of Science Core Collection

((((((((((TS=(“Health auxiliar*” OR “peer group*” OR “health visitor*” OR doula* OR “Allied health personnel” OR “Community health worker*” OR paramedic* OR paraprofessional* “allied health worker*” OR “support worker*” OR “support worker*” OR “home health aide*” OR montrice* OR linkworker* OR “link worker*” OR “barefoot doctor*” OR “expert patient*” OR “health promoter*” OR “health extension worker*” OR “mentor mother*”)) OR TS=((lay OR voluntary OR volunteer* OR untrained OR unlicensed OR "non-professional" OR nonprofessional OR informal OR "non-formal") NEAR/5 (worker* OR visitor* OR attendant* OR aide* OR support* OR person* OR helper* OR carer* OR caregiver* OR "care giver" OR "care givers" OR consultant* OR assistant* OR staff OR visit* OR midwife OR midwives OR provider* OR practitioner*))) OR TS=(trained NEAR/3 (volunteer* OR health NEAR/0 worker* OR mother*))) OR TS=((communit* OR village* OR frontline*) NEAR/3 ((health NEAR/0 worker*) OR (health NEAR/0 care NEAR/0 worker*) OR (healthcare NEAR/0 worker*) OR distributor* OR worker* OR provider*))) OR TS=(communit* NEAR/3 (volunteer* OR aide* OR support))) OR TS=((birth OR childbirth OR labor OR labour) NEAR/1 (attendant* OR assistant*))) OR TS=((lay OR peer) NEAR/1 (volunteer* OR mentor* OR counsel* OR support OR intervention*))) OR TS=((church NEAR/0 based NEAR/3 (intervention* OR program* OR counsel*)))) OR TS=(((health OR healthcare) NEAR/1 outreach))) OR TS=((home NEAR/1 (care OR aide* OR nursing OR support OR intervention* OR treatment* OR visit*)))) OR TS=(((care OR aide* OR nursing OR support OR intervention* OR treatment* OR visit*) NEAR/3 (lay OR volunteer* OR voluntary)))) OR TS=((Auxiliary NEAR/3 (worker* OR nurse* OR midwives OR midwife)))
AND

((((TI=(economic* OR cost OR costs OR costly OR costing OR price OR prices OR pricing OR expenditure OR expenditures OR expense OR expenses OR financial OR finance OR finances OR financed) OR AK=(economic* OR cost OR costs OR costly OR costing OR price OR prices OR pricing OR expenditure OR expenditures OR expense OR expenses OR financial OR finance OR finances OR financed) OR AB=(cost* NEAR/2 (effective* OR utilit* OR benefit* OR minimi* OR analy* OR outcome OR outcomes)) OR AK=(cost* NEAR/2 (effective* OR utilit* OR benefit* OR minimi* OR analy* OR outcome OR outcomes))) OR TS=((value NEAR/2 (money OR monetary)))) OR TS=("economic model*")) OR TS=((decision* NEAR/2 (tree* OR analy* OR model*)))) OR TS=(("Disability-Adjusted Life Years" OR DALYS OR "quality-adjusted life years" OR QALYS OR "Return on investment" OR "multiple criteria decision analysis" or MCDA OR "cost-benefit analysis" OR "Economic evaluation*" OR "economic analys*" OR "Financial planning" OR "financial management"))
AND
(Afghanistan OR Burundi OR "Burkina Faso" OR "Central African Republic" OR Congo OR Eritrea OR Ethiopia OR Guinea OR Gambia OR Guinea-Bissau OR Liberia OR Madagascar OR Mali OR Mozambique OR Malawi OR Niger OR "North Korea" OR Rwanda OR Sudan OR "Sierra Leone" OR Somalia OR "South Sudan" OR "Syrian Arab Republic" OR Chad OR Togo OR Uganda OR Yemen OR Zambia OR Angola OR Benin OR Bangladesh OR Bolivia OR Bhutan OR "cote d'ivoir*" OR "cote d' ivoir*" OR "cote divoir*" OR "cote d ivoir*" OR "ivory coast*" OR Cameroon OR Comoros OR "Cabo Verde" OR Djibouti OR Algeria OR Egypt OR Micronesia OR Ghana OR Honduras OR Haiti OR Indonesia OR India OR Iran OR Kenya OR Kyrgyzstan OR Cambodia OR Kiribati OR Laos OR Lebanon OR "Sri Lanka" OR Lesotho OR Morocco OR Myanmar OR Burma OR Mongolia OR Mauritania OR Nigeria OR Nicaragua OR Nepal OR Pakistan OR Philippines OR "Papua New Guinea" OR "West Bank" OR Gaza OR Senegal OR "Solomon Islands" OR "El Salvador" OR "Sao Tome and Principe" OR Eswatini OR Swaziland OR Tajikistan OR Timor-Leste OR Tunisia OR Tanzania OR Ukraine OR Uzbekistan OR Vietnam OR Vanuatu OR Samoa OR Zimbabwe OR Afghan* OR Burundian* OR Burkinabe* OR "Central African*" OR Congolese OR Eritrean* OR Ethiopian* OR Guinean* OR Gambian* OR Guinea-Bissauan* OR Liberian* OR Malagasy* OR Malian* OR Mozambican* OR Malawian* OR Nigerien* OR "North Korean*" OR Rwandan* OR Sudanese OR "Sierra Leonean*" OR Somali* OR Syrian OR Chadian* OR Togolese OR Ugandan* OR Yemeni* OR Zambian* OR Angolan* OR Benin* OR Bangladeshi* OR Bolivian* OR Bhutanese* OR Ivorian* OR Cameroonian* OR Comorian* OR Cape Verdean* OR Djiboutian* OR Algerian* OR Egyptian*

OR Micronesian* OR Ghanaian* OR Honduran* OR Haitian* OR Indonesian* OR (Indian* NOT indiana) OR Iranian* OR Kenyan* OR Kyrgyz* OR kirgiz* OR kirghiz* OR Cambodian* OR Kiribatian* OR Laotian* OR Lebanese OR "Sri Lankan*" OR Basotho* OR Moroccan* OR Burmese OR Mongolian* OR Mauritanian* OR Nigerian* OR Nicaraguan* OR Nepalese OR Pakistani* OR Filipino* OR "Papua New Guinean*" OR Palestinian* OR Senegalese OR "Solomon Islander*" OR Salvadoran* OR Salvadorian* OR Sao Tomean* OR Eswatini* OR swazi* OR swati* OR Tajik* OR Timorese OR Tunisian* OR Tanzanian* OR Ukrainian* OR Uzbek* OR Vietnamese OR Vanuatuan* OR Samoan* OR Zimbabwean* OR africa* OR "arab* countr*" OR "middle east*" OR "global south" OR sahara* OR subsahara* OR magreb* OR maghrib* OR "west indies*" OR caribbean* OR "central america*" OR "latin america*" OR "south america*" OR "central asia*" OR "north asia*" OR "northern asia*" OR "southeastern asia*" OR "south eastern asia*" OR "southeast asia*" OR "south east asia*" OR "west asia*" OR "western asia*" OR "east europe*" OR "eastern europe*" OR "developing countr*" OR "developing nation*" OR "developing population*" OR "developing world" OR "less developed countr*" OR "less developed nation*" OR "less developed world" OR "lesser developed countr*" OR "lesser developed nation*" OR "lesser developed world" OR "under developed countr*" OR "under developed nation*" OR "under developed world" OR "underdeveloped countr*" OR "underdeveloped nation*" OR "underdeveloped world" OR "middle income countr*" OR "middle income nation*" OR "middle income population*" OR "low income countr*" OR "low income nation*" OR "low income population*" OR "lower income countr*" OR "lower income nation*" OR "lower income population*" OR "underserved countr*" OR "underserved nation*" OR "underserved population*" OR "under served population*" OR "under served nation*" OR "deprived countr*" OR "deprived population*" OR "high burden countr*" OR "high burden nation*" OR "countdown countr*" OR "countdown nation*" OR "poor countr*" OR "poor nation*" OR "poor population*" OR "poor world" OR "poorer countr*" OR "poorer nation*" OR "poorer population*" OR "poorer world" OR "developing econom*" OR "less developed econom*" OR "underdeveloped econom*" OR "under developed econom*" OR "middle income econom*" OR "low income econom*" OR "lower income econom*" OR "low gdp" OR "low gnp"
AND
2023 or 2022 or 2021 or 2020 or 2019 or 2018 or 2017 or 2016 or 2015 (Publication Years)

Scopus

(TITLE-ABS-KEY ({Health auxiliary} OR {Health auxiliaries} OR {peer group} OR {peer groups} OR {health visitor} OR {health visitors} OR doula* OR {Allied health personnel} OR {Community health worker} OR {Community health workers} OR paramedic* OR paraprofessional* OR {allied health worker} OR {allied health workers} OR {support worker} OR {support workers} OR {home health aide} OR {home health aides} OR montrice* OR linkworker* OR {link worker} OR {link workers} OR {barefoot doctor} OR {barefoot doctors} {expert patient} OR {expert patients} OR {health promoter} OR {health promoters} OR {health extension worker} OR {health extension workers} OR {mentor mother} OR {mentor mothers}) OR TITLE-ABS-KEY ((lay OR voluntary OR volunteer* OR untrained OR unlicensed OR "non professional" OR nonprofessional* OR informal OR "non-formal") W/5 (worker* OR visitor* OR attendant* OR aide* OR support* OR person* OR helper* OR carer* OR caregiver* OR "care giver" OR "care givers" OR consultant* OR assistant* OR staff OR visit* OR midwife OR midwives OR provider* OR practitioner*)) OR TITLE-ABS-KEY (trained W/3 (volunteer* OR "health worker" OR "health workers" OR mother*)) OR TITLE-ABS-KEY ((communit* OR village* OR frontline*) W/3 ("health worker" OR "health workers" OR "health care worker" OR "health care workers" OR "healthcare worker" OR "healthcare workers" OR distributor* OR worker* OR provider*)) OR TITLE-ABS-KEY (communit* W/3 (volunteer* OR aide* OR support)) OR TITLE-ABS-KEY ((birth OR childbirth OR labor OR labour) W/1 (attendant* OR assistant*)) OR TITLE-ABS-KEY ((lay OR peer) W/1 (volunteer* OR mentor* OR counsel* OR support OR intervention*)) OR TITLE-ABS-KEY ("church based" W/3 (intervention* OR program* OR counsel*)) OR TITLE-ABS-KEY ((health OR healthcare) W/1 outreach) OR TITLE-ABS-KEY (home W/1 (care OR aide* OR nursing OR support OR intervention* OR treatment* OR visit*)) OR TITLE-ABS-KEY ((care OR aide* OR nursing OR support OR intervention* OR treatment* OR visit*) W/3 (lay OR volunteer* OR voluntary)) OR TITLE-ABS-KEY (auxiliary W/3 (worker* OR nurse* OR midwives OR midwife))) AND (TITLE (economic* OR cost OR costs OR costly OR costing OR price OR prices OR pricing OR expenditure OR expenditures OR expense OR expenses OR financial OR finance OR finances OR financed) OR KEY (economic* OR cost OR costs OR costly OR costing OR price OR prices OR pricing OR expenditure OR expenditures OR expense OR expenses OR financial OR finance OR finances OR financed) OR ABS (cost* W/2 (effective* OR utilit* OR benefit* OR minimi* OR analy* OR outcome OR outcomes)) OR KEY (cost* W/2 (effective* OR utilit* OR benefit* OR minimi* OR analy* OR outcome OR outcomes)) OR TITLE-ABS-KEY (value W/2 (money OR monetary)) OR TITLE-ABS-KEY ("economic model" OR "economic models") OR TITLE-ABS-KEY (decision* W/2 (tree* OR analy* OR model*)) OR TITLE-ABS-KEY ("Disability-Adjusted Life Years" OR dalys OR "quality-adjusted life years" OR qalys OR "Return on investment" OR "multiple criteria decision analysis" OR mcda OR "cost-benefit analysis" OR "Economic evaluation" OR "Economic evaluations" OR "economic analysis" OR "economic analyses" OR "Financial planning" OR "financial management")) AND (TITLE-ABS-KEY ((afghanistan OR burundi OR "Burkina Faso" OR "Central African Republic" OR congo OR eritrea OR ethiopia OR guinea OR gambia OR guinea-bissau OR liberia OR madagascar OR mali OR mozambique OR malawi OR niger OR "North Korea" OR rwanda OR sudan OR "Sierra Leone" OR somalia OR "South Sudan" OR "Syrian Arab Republic" OR chad OR togo OR uganda OR yemen OR zambia OR angola OR benin OR bangladesh OR bolivia OR bhutan OR "cote d'ivoir*" OR "cote d' ivoir*" OR "cote divoir*" OR "cote d ivoir*" OR "ivory coast*" OR cameroon OR comoros OR "Cabo Verde" OR djibouti OR algeria OR egypt OR micronesia OR ghana OR honduras OR haiti OR indonesia OR india OR iran OR kenya OR kyrgyzstan OR cambodia OR kiribati OR laos OR lebanon OR "Sri Lanka" OR lesotho OR morocco OR myanmar OR burma OR mongolia OR mauritania OR nigeria OR nicaragua OR nepal OR pakistan OR philippines OR "Papua New Guinea" OR "West Bank" OR gaza OR senegal OR "Solomon Islands" OR "El Salvador" OR "Sao Tome and Principe" OR eswatini OR swaziland OR tajikistan OR timor-leste OR tunisia OR tanzania OR ukraine OR uzbekistan OR vietnam OR vanuatu OR samoa OR zimbabwe OR afghan* OR burundian* OR burkinabe* OR "Central African*" OR congolese OR eritrean* OR ethiopian* OR guinean* OR gambian* OR guinea-bissauan* OR liberian* OR malagasy* OR malian* OR mozambican* OR malawian* OR nigerien* OR "North Korean*" OR rwandan*

OR sudanese OR "Sierra Leonean*" OR somali* OR syrian OR chadian* OR togolese OR ugandan* OR yemeni* OR zambian* OR angolan* OR benin* OR bangladeshi* OR bolivian* OR bhutanese* OR ivorian* OR cameroonian* OR comorian* OR cape AND verdean* OR djiboutian* OR algerian* OR egyptian* OR micronesian* OR ghanaian* OR honduran* OR haitian* OR indonesian* OR (indian* AND not AND indiana) OR iranian* OR kenyan* OR kyrgyz* OR kirgiz* OR kirghiz* OR cambodian* OR kiribatian* OR laotian* OR lebanese OR "Sri Lankan*" OR basotho* OR moroccan* OR burmese OR mongolian* OR mauritanian* OR nigerian* OR nicaraguan* OR nepalese OR pakistani* OR filipino* OR "Papua New Guinean*" OR palestinian* OR senegalese OR "Solomon Islander*" OR salvadoran* OR salvadorian* OR sao AND tomean* OR eswatini* OR swazi* OR swati* OR tajik* OR timorese OR tunisian* OR tanzanian* OR ukrainian* OR uzbek* OR vietnamese OR vanuatuan* OR samoan* OR zimbabwean* OR africa* OR "arab* countr*" OR "middle east*" OR "global south" OR sahara* OR subsahara* OR magreb* OR maghrib* OR "west indies*" OR caribbean* OR "central america*" OR "latin america*" OR "south america*" OR "central asia*" OR "north asia*" OR "northern asia*" OR "southeastern asia*" OR "south eastern asia*" OR "southeast asia*" OR "south east asia*" OR "west asia*" OR "western asia*" OR "east europe*" OR "eastern europe*" OR "developing countr*" OR "developing nation*" OR "developing population*" OR "developing world" OR "less developed countr*" OR "less developed nation*" OR "less developed world" OR "lesser developed countr*" OR "lesser developed nation*" OR "lesser developed world" OR "under developed countr*" OR "under developed nation*" OR "under developed world" OR "underdeveloped countr*" OR "underdeveloped nation*" OR "underdeveloped world" OR "middle income countr*" OR "middle income nation*" OR "middle income population*" OR "low income countr*" OR "low income nation*" OR "low income population*" OR "lower income countr*" OR "lower income nation*" OR "lower income population*" OR "underserved countr*" OR "underserved nation*" OR "underserved population*" OR "under served population*" OR "under served nation*" OR "deprived countr*" OR "deprived population*" OR "high burden countr*" OR "high burden nation*" OR "countdown countr*" OR "countdown nation*" OR "poor countr*" OR "poor nation*" OR "poor population*" OR "poor world" OR "poorer countr*" OR "poorer nation*" OR "poorer population*" OR "poorer world" OR "developing econom*" OR "less developed econom*" OR "underdeveloped econom*" OR "under developed econom*" OR "middle income econom*" OR "low income econom*" OR "lower income econom*" OR "low gdp" OR "low gnp" OR "low gross domestic" OR "low gross national" OR "lower gdp" OR "lower gnp" OR "lower gross domestic" OR "lower gross national" OR lmic OR lmics OR "third world" OR "lami countr*" OR "transitional countr*" OR "emerging econom*" OR "emerging nation*"))) AND PUBYEAR > 2014 AND PUBYEAR < 2024

LILACS

tw:'Health auxiliary' OR tw:'Health auxiliaries' OR tw:'peer group' OR tw:'peer groups' OR tw:'health visitor' OR tw:'health visitors' OR tw:doula* OR tw:'Allied health personnel' OR tw:'Community health worker' OR tw:'Community health workers' OR tw:paramedic* OR tw:paraprofessional* OR tw:'allied health worker' OR tw:'allied health workers' OR tw:'support worker' OR 'tw:support workers' OR tw:'home health aide' OR tw:'home health aides' OR tw:montrice* OR tw:linkworker* OR tw:'link worker' OR tw:'barefoot doctor' OR tw:'barefoot doctors' OR tw:'expert patient' OR tw:'expert patients' OR tw:health promoter* OR tw:'health extension worker' OR tw:'health extension workers' OR tw:'mentor mother' OR tw:'mentor mothers'OR tw:carer* OR tw:caregiver* OR tw:'care giver' OR tw:midwife OR tw:midwives OR tw:paraprofessional* OR tw:paramedic* OR tw:'allied health worker' OR tw:'allied health workers' OR tw:'support worker' OR tw:'support workers' OR tw:'trained volunteer' OR tw:'trained volunteers' OR tw:'trained health worker' OR tw:'trained health workers' OR tw:'trained mother' OR tw:'trained mothers' OR tw:'community health care worker' OR tw:'community health care workers' OR tw:'community healthcare worker' OR tw:'community healthcare workers' OR tw:'village health worker' OR tw:'village health workers' OR tw:'frontline provider' OR tw:'frontline

providers' OR tw:'community volunteer' OR tw:'community volunteers' OR tw:'community aide' OR tw:'community aides' OR tw:'community support' OR tw:'birth attendant' OR tw:'birth attendants' OR tw:'birth assistant' OR tw:'birth assistants' OR tw:'childbirth attendant' OR tw:'childbirth attendants' OR tw:'childbirth assistant' OR tw:'childbirth assistants' OR tw:'labor attendant' OR tw:'labor attendants' OR tw:'labor assistant' OR tw:'labor assistants' OR tw:'labour attendant' OR tw:'labour attendants' OR tw:'labour assistant' OR tw:'labour assistants' OR tw:'lay volunteer' OR tw:'lay volunteers' OR tw:'lay counselor' OR tw:'lay counselors' OR tw:'peer volunteer' OR tw:'peer volunteers' OR tw:'peer support' OR tw:'peer mentor' OR tw:'peer mentors' OR tw:'church based intervention' OR tw:'church based interventions' OR tw:'church based program' OR tw:'church based programs' OR tw:'church based counseling' OR tw:'health outreach' OR tw:'healthcare outreach' OR tw:'home care' OR tw:'home aide' OR tw:'home visit' OR tw:'home visits' OR tw:'volunteer nurse' OR tw:'volunteer nurses' OR tw:'auxiliary worker' OR tw:'auxiliary workers' OR tw:'auxiliary nurse' OR tw:'auxiliary nurses' OR tw:'health promoter' OR tw:'health promoters'
