# Supplementary material for: Costs and cost-effectiveness of community health worker programs on reproductive, maternal, newborn and child health in low- and middle-income countries (2015–2024): A scoping review
Source: PLOS Glob Public Health. 2026 Jan 22;6(1):e0004893. doi: 10.1371/journal.pgph.0004893 (PMC12826514; doi:10.1371/journal.pgph.0004893)
Supplement: S2 File — (PDF) [file pgph.0004893.s006.pdf]

Reflexivity Checklist

Adapted from Morton et al., Consensus statement on measures to promote equitable authorship in the publication of research from international partnerships.

**Study conceptualisation**	
How does this study address local research and policy priorities?	This study (including the aims and objectives) was formulated in partnership with key stakeholders from the Community Health Impact Coalition (CHIC) a network of key stakeholders working across 40 countries to strengthen community health systems. Increasingly, task shifting and task sharing for NCDs through the involvement of CHWs is occurring. This study set out to better understand the costs, cost-effectiveness and affordability of such efforts.
How were local researchers involved in study design?	The study involves over 20 co-authors who were pivotally involved in the design, implementation and writing of this scoping review. 8 of the co-authors are from LMICs. We also ran the study protocol past a larger group of over 70 researchers from LMIC settings and a group of 10 CHWs to ensure the research questions were aligned with local priorities in LMICs.
**Research management**	
How has funding been used to support the local research team(s)?	Whilst we did not have any specific funding for this study the senior members of the team have supported more junior members of the research team with pro-bono mentoring in research methods and writing skills.
**Data acquisition and analysis**	
How are research staff who conducted data collection acknowledged?	Either as co-authors or explicitly by name in the Acknowledgements section.
How have members of the research partnership been provided with access to study data?	All data is open access to the entire team via a shared drive.
How were data used to develop analytical skills within the partnership?	The senior members of the team have supported more junior members of the research team with developing analytical skills especially in relation to economic evaluation.
**Data interpretation**	

How have research partners collaborated in interpreting study data?	All partners were involved in the shaping of the discussion section (including CHW partners)
**Drafting and revising for intellectual content**	
How were research partners supported to develop writing skills?	Through iterative feedback on the shared document.
How will research products be shared to address local needs?	The research findings will be shared via press-releases through the CHIC network. We will also share the findings via video which will be translated into several key languages and also through community workshops and through our network of CHWs who attend monthly meetings with CHIC.
**Authorship**	
How is the leadership, contribution and ownership of this work by LMIC researchers recognised within the authorship?	As joint co-authors and in the reflexivity statement in the manuscript.
How have early career researchers across the partnership been included within the authorship team?	Yes
How has gender balance been addressed within the authorship?	Yes. The senior author is female and of the 20 co-authors there is a majority female authorship.
**Training**	
How has the project contributed to training of LMIC researchers?	Yes.
**Infrastructure**	
How has the project contributed to improvements in local infrastructure?	N/A (done remotely as a desk scoping review).
**Governance**	
What safeguarding procedures were used to protect local study participants and researchers?	This was a scoping review conducted remotely however all participants adhered to the CHIC Research safeguarding procedures and good practice guidance.
